# Anisotropic Photoluminescence of Poly(3-hexyl thiophene) and Their Composites with Single-Walled Carbon Nanotubes Highly Separated in Metallic and Semiconducting Tubes

**DOI:** 10.3390/molecules26020294

**Published:** 2021-01-08

**Authors:** Mihaela Baibarac, Grigory Arzumanyan, Monica Daescu, Adelina Udrescu, Kahramon Mamatkulov

**Affiliations:** 1 Optical Processes in Nanostructure Materials Laboratory, National Institute of Materials Physics, Atomistilor Str. 405 A, 77125 Magurele, Romania; monica.daescu@infim.ro (M.D.); adelina.udrescu@infim.ro (A.U.); 2 Neutron Physics, Joint Institute for Nuclear Research Laboratory, 6 Joliot-Curie Street, 141980 Dubna, Russia; arzuman@jinr.ru (G.A.); hero170184@mail.ru (K.M.)

**Keywords:** anisotropic photoluminescence, photoluminescence quenching process, SERS spectroscopy, IR spectroscopy

## Abstract

In this work, the effect of the single-walled carbon nanotubes (SWNTs) as the mixtures of metallic and semiconducting tubes (M + S-SWNTs) as well as highly separated semiconducting (S-SWNTs) and metallic (M-SWNTs) tubes on the photoluminescence (PL) of poly(3-hexyl thiophene) (P3HT) was reported. Two methods were used to prepare such composites, that is, the chemical interaction of the two constituents and the electrochemical polymerization of the 3-hexyl thiophene onto the rough Au supports modified with carbon nanotubes (CNTs). The measurements of the anisotropic PL of these composites have highlighted a significant diminution of the angle of the binding of the P3HT films electrochemical synthetized onto Au electrodes covered with M + S-SWNTs. This change was attributed to metallic tubes, as was demonstrated using the anisotropic PL measurements carried out on the P3HT/M-SWNTs and P3HT/S-SWNTs composites. Small variations in the angle of the binding were reported in the case of the composites prepared by chemical interaction of the two constituents. The proposed mechanism to explain this behavior took into account the functionalization process of CNTs with P3HT. The experimental arguments of the functionalization process of CNTs with P3HT were shown by the UV-VIS-NIR and FTIR spectroscopy as well as surface-enhanced Raman scattering (SERS). A PL quenching process of P3HT induced both in the presence of S-SWNTs and M-SWNTs was reported, too. This process origins in the various de-excitation pathways which can be developed considering the energy levels diagram of the two constituents of each studied composite.

## 1. Introduction

Much effort has been made in recent years regarding the fundamental properties of carbon nanoparticles such as carbon nanotubes and carbon quantum dots for applications in the field of energy storage, health and photovoltaic devices [1,2,3,4]. The applications of the composites based on poly(3-hexyl thiophene) (P3HT) and carbon nanotubes (CNTs) in the photovoltaic devices [5,6] and solar cells [7,8] involved a better understanding of the synthesis protocols as well as the structure-properties relationship [9]. The main synthesis protocols used in the CNTs-based composites field were the chemical interaction of the two constituents, chemical polymerization of monomer in the presence of CNTs and the electrochemical polymerization of monomer onto working electrodes modified with the CNTs [10]. In this work for the particular case of the P3HT/CNT composites, the chemical interaction of P3HT with CNTs and the electrochemical polymerization of 3-hexylthiophene (3HT) onto the rough gold electrodes modified with CNTs will be used. Three type of products were reported to result from these synthesis methods in the case of the composites based on conducting polymers (CPs) and CNTs: CPs doped with the CNTs anion radicals, CPs covalently functionalized CNTs and CPs non-covalently functionalized CNTs [10]. Generally, the information concerning the reaction products resulted from the three synthesis methods were obtained by Raman scattering, UV-VIS-NIR and Fourier transform infrared (FTIR) spectroscopy X-ray photoelectron spectroscopy and high-resolution transmission microscopy [11]. In this work, arguments concerning the type of the P3HT/CNTs composite will be shown by the studies of the surface-enhanced Raman scattering (SERS), FTIR and UV-VIS-NIR spectroscopy. Regardless of the synthesis method used for single-walled carbon nanotubes (SWNTs), that is, laser ablation, chemical vapor deposition, catalytic growth in flame and electrical arc discharge, a mixture of metallic and semiconducting tubes (M + S-SWNTs) was obtained [12]. Various methods were developed to purification and to separate the metallic and semiconducting SWNTs, these leading to the obtaining of highly separated metallic tubes (98%, M-SWNTs) and semiconducting tubes (99%, S-SWNTs) [13,14]. A consequence of this progress has been a better understanding of the fundamental processes of these nanomaterials, as well as their role in applied researchers. In this context, a sustained effort was carried out in the last years in order to evidence the influence of the metallic and semiconducting tubes on both the physical properties, for example, photoluminescence (PL) [15] and applications field, for example, sensors [16], organic light-emitting diode [17], organic photovoltaic [18] and son on. Often, a CPs PL quenching process was reported to be induced by M + S-SWNTs [15,19]. Depending on the energy levels diagrams of the CPs and M + S-SWNTs, the CPs PL quenching process was reported to be induced either by semiconducting tubes, either of metallic tubes [15,19]. In this work, the influence of M + S-SWNTs, S-SWNTs and M-SWNTs on the P3HT PL, when samples are as solution and film deposited onto rough gold supports, will be reported, too. In the case of the film samples, the influence of their preparation methods will also be highlighted. A little studied issue is the anisotropic PL of the CP/CNT composites, at present being published only one article focused on the P3HT/M + S-SWNTs fibers obtained by a wet-process in the membrane of the type anodized alumina oxide [20]. After the best knowledge of authors, until now a comparative study concerning the influence of M + S-SWNTs, S-SWNTs and M-SWNTs on the anisotropic PL of CPs, which shows the role of the semiconducting and metallic CNTs, respectively, in the anisotropic PL of P3HT has not been reported. A novelty element of this work consists in the highlighting of the changes induced by S-SWNTs and M-SWNTs on the anisotropic PL of P3HT. In addition, the variations induced by the three types of SWNTs on the P3HT anisotropic PL as depending on the method of the interaction of the two constituents, that is, chemical or electrochemical, used to prepare the P3HT/CNTs composites, will be shown, too.

## 2. Results and Discussion

### 2.1. IR Spectra of P3HT and Its Composites with CNTs Prepared by Chemical and Electrochemical Methods

**Figure 1:**
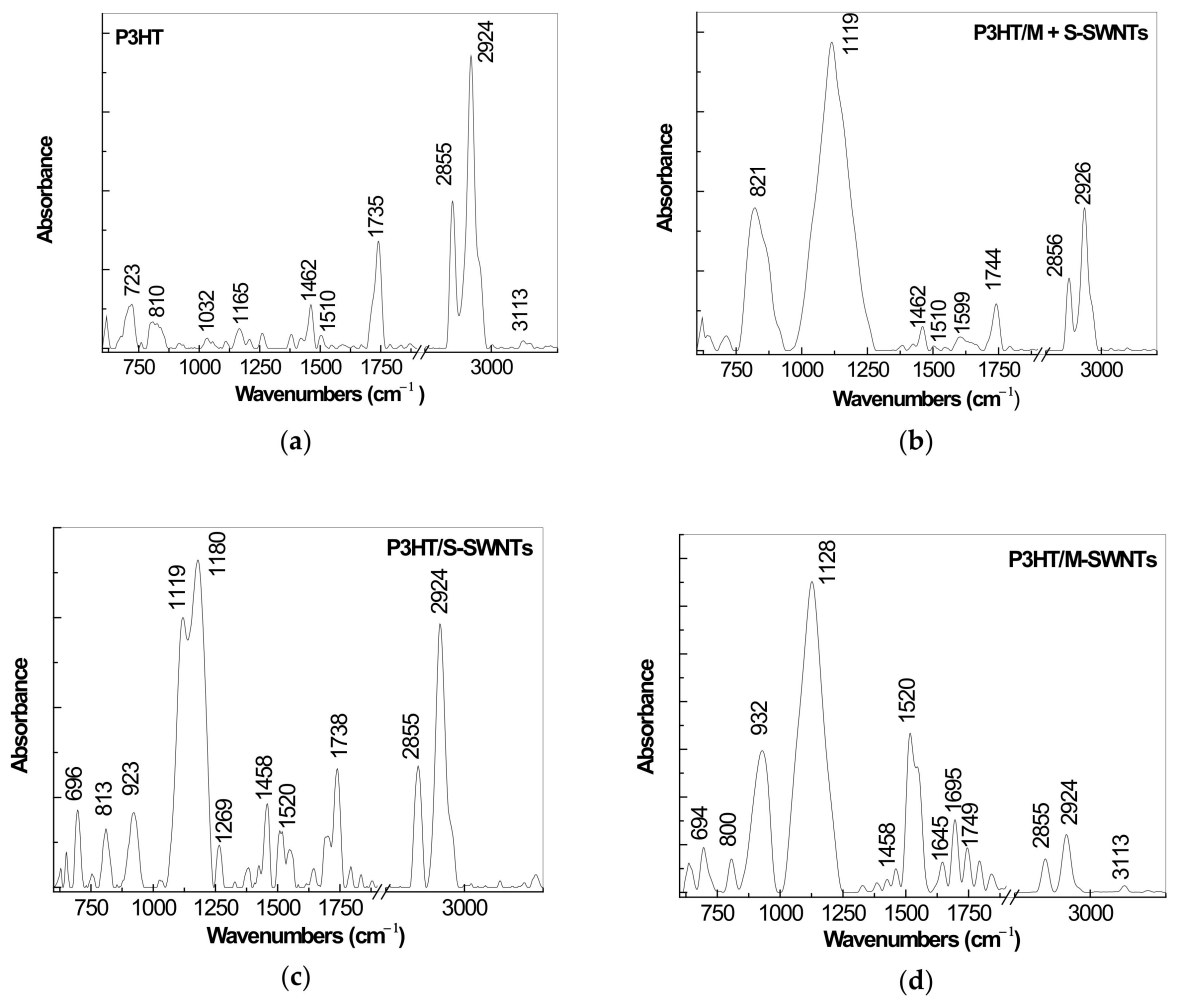
Fourier transform infrared (FTIR) spectra of P3HT (**a**) and their composites of the type P3HT/M + S- single-walled carbon nanotubes (SWNTs) (**b**), P3HT/S-SWNTs (**c**) and P3HT/M-SWNTs (**d**), obtained by chemical interaction of the two constituents in semi-aqueous solution and successively deposited as films onto the rough Au supports.

**Scheme 1:**
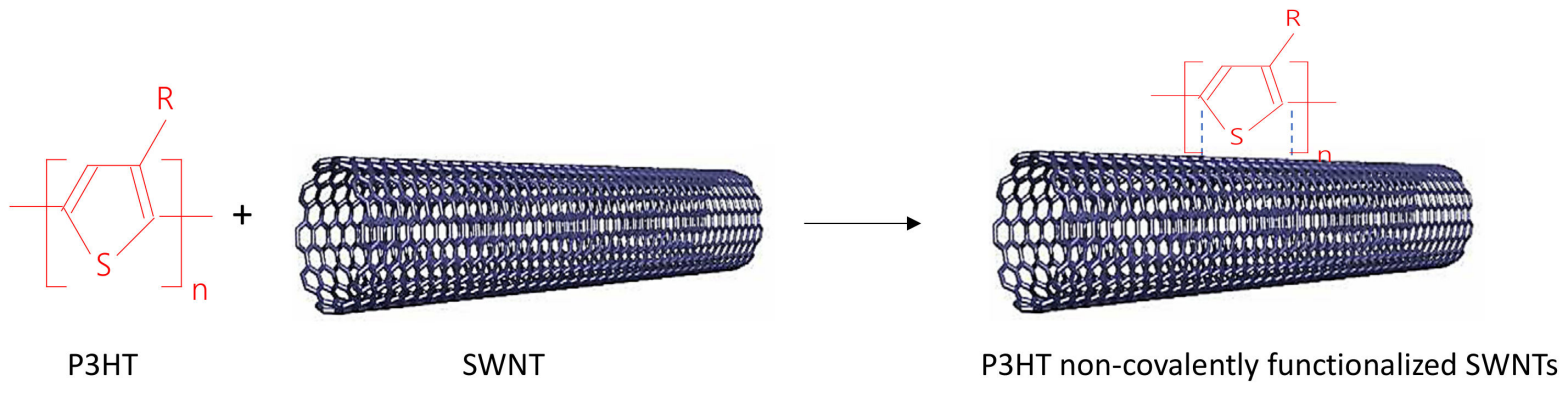
The interaction of P3HT with SWNTs of the type M-SWNTs or S-SWNTs, when the chemical method is used for the obtaining P3HT non-covalently functionalized SWNTs. The blue dotted lines highlight π-π stacking interactions between the two constituents. R corresponds to the hexyl group.

Figure 1a shows the main IR bands of P3HT, which are peaked at 723, 810, 1032, 1165, 1462, 1510, 1735, 2854, 2924 and 3113 cm^−1^, they being assigned to the following vibrational modes: rocking CH_2_, out of plane bending C-S in thiophene ring α, α’ disubstituted, aromatic C-H stretching, thiophene ring, asymmetrical C=C in thiophene ring, C=O stretching, symmetric C-H stretching in CH_2_ moieties, C-H in hexyl group and sp^2^ C-H stretching in aromatic compounds, respectively [8,21]. The IR bands at 617 and 1381 cm^−1^ were attributed to the vibrational modes of the distorted parts of polymer and the deformation of -CH_3_ group belonging to the defects present in the P3HT macromolecular chains, respectively [22,23]. Chemical interaction of P3HT with M + S-SWNTs, S-SWNTs and M-SWNTs induces the following variations in Figure 1: (i) the appearance of an IR band, situated in the spectral range 1000–1250 cm^−1^, which belongs to SWNTs. According to early studies of FTIR spectroscopy, in the spectral range 1000–1200 cm^−1^, SWNTs show three IR bands peaked at 1080–1095, 1164–1173 and 1197 cm^−1^ assigned to the 2nd order 2E_9_ (10, 10) + 2E_19_ (15, 0) + 2E_10_ (14, 7) vibrational modes [24]; (ii) a down-shift of the IR band from 1462 cm^−1^ (Figure 1a) to 1458 cm^−1^ (Figure 1c,d); (iii) a change of the ratio between the absorbance of the IR band peaked at 1462–1458 and 2924–2926 cm^−1^ from 0.15 (Figure 1a) to 0.17 (Figure 1b), 0.31 (Figure 1c) and 0.4 (Figure 1d); and (iv) the change of the ratio between the absorbance of the IR bands peaked at 1462–1458 and 1510–1520 cm^−1^ form 3.96 (Figure 1a) to 5.45 (Figure 1b), 1.53 (Figure 1c) and 0.14 (Figure 1d). Taking into account that the IR band at 1510 cm^−1^ assigned to the asymmetrical C=C vibrational mode in thiophene ring increases in absorbance as increasing of the conjugation length of P3HT [22], above variations suggest that the adsorption of P3HT onto SWNTs surface that involves π-π stacking interactions between the C=C bonds from basal plane of graphitic lattice of SWNTs and the C=C bonds of thiophene ring [25], as shown in Scheme 1, when a non-covalently functionalization process of M-SWNTs and S-SWNTs with macromolecular compound takes place. A consequence of these π-π bonds is the appearance of some hindrance steric effects that are induced to the C-H bonds of thiophene ring and hexyl group.

**Figure 2:**
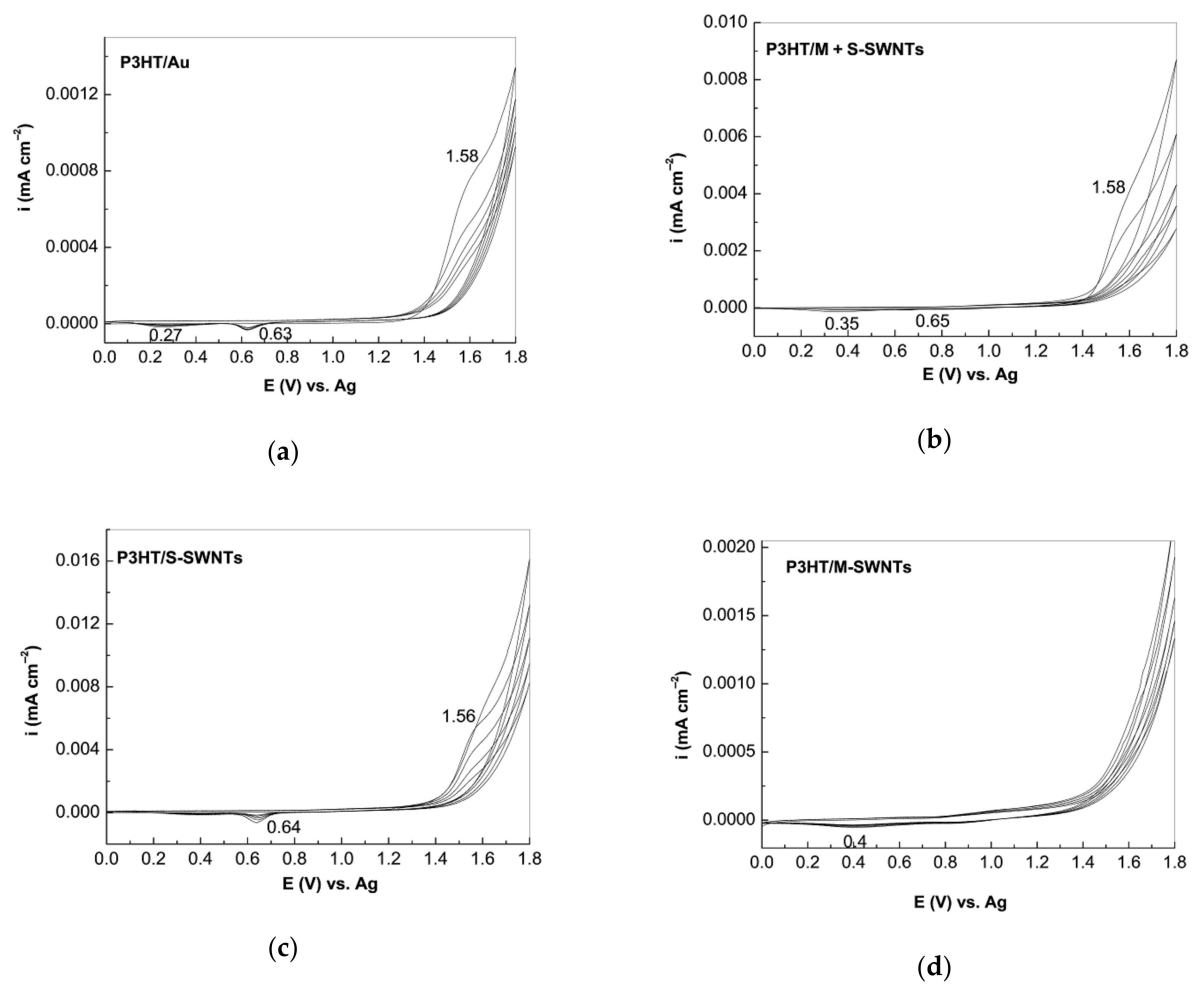
The first 30 cyclic voltammograms recorded during the polymerization of 3HT in the presence of [BMIM]PF_6_ onto the Au electrode before (**a**) and after its modification with CNTs of the type M + S-SWNTs (**b**), S-SWNTs (**c**) and M-SWNTs (**d**). The thickness of CNTs films was of 50 nm.

Figure 2a shows the first 30 cyclic voltammograms recorded during electrochemical polymerization of 3HT onto the blank Au electrode. As increasing the cycles number, an increase in the anodic and cathodic currents densities takes place. This result is due to the 3HT electrochemical polymerization when is generated an increasing amount of polymer on the working electrode surface. The oxidation reaction of 3HT takes place in the potential range (+1.3; +1.8) V, where is observed the anodic peak characterized by the potential of +1.58 V vs. Ag. Two cathodic peaks are noted during the scanning of the potential from +1.8 V to 0 V, having the maxima potential of +0.27 and +0.63 V. Considering the ratio between the current densities of the anodic and cathodic peaks, which is different of one and that the potential of separation of the anodic and cathodic peaks is different of 56.5/*n* mV, where *n* corresponds to the electron number involved in electrochemical process, we conclude that processes that take place at the electrode/electrolyte interface have an irreversible character. Figure 2b,c show a similar behavior with the only difference that the anodic and cathodic current densities are higher in contract with those highlighted in Figure 2a. In Figure 2d one observes that the oxidation reaction of 3HT is developed in the potential range (+1.3; +1.8) V but an anodic peak is not highlighted as in the case of Figure 2a–c.

**Figure 3:**
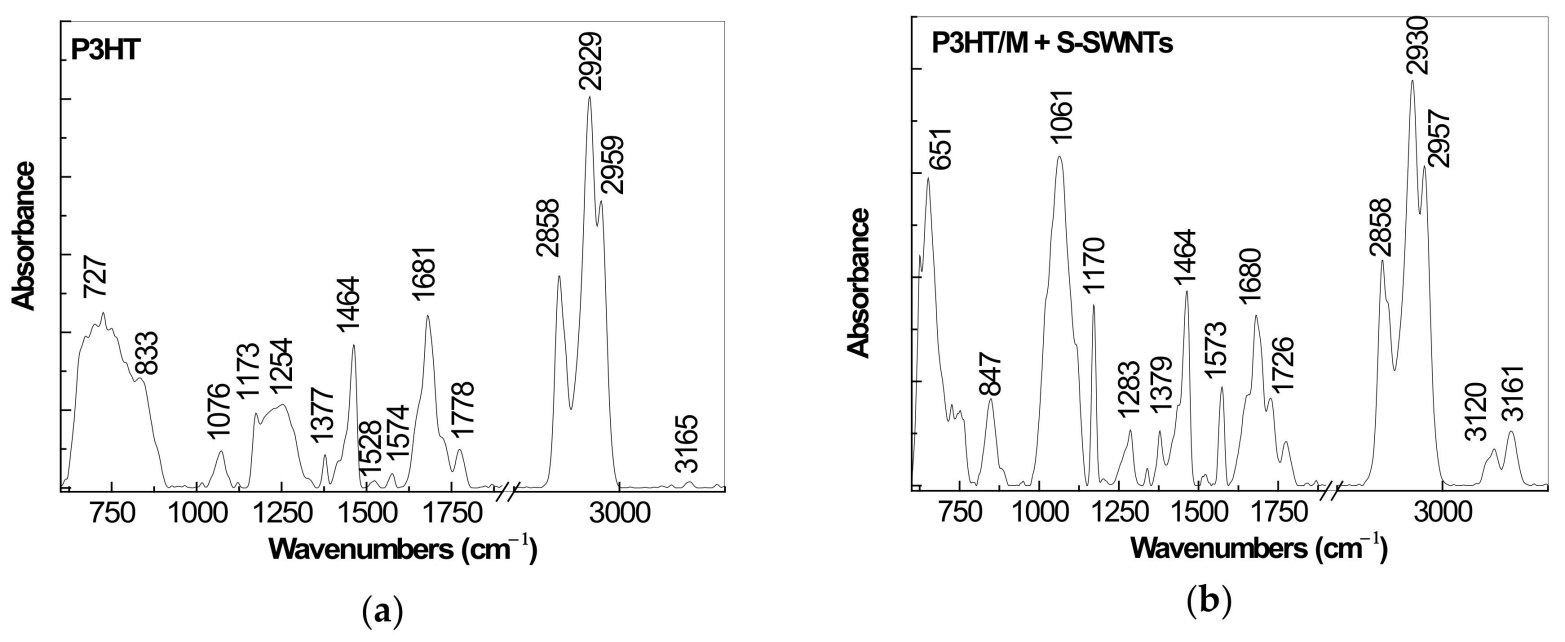

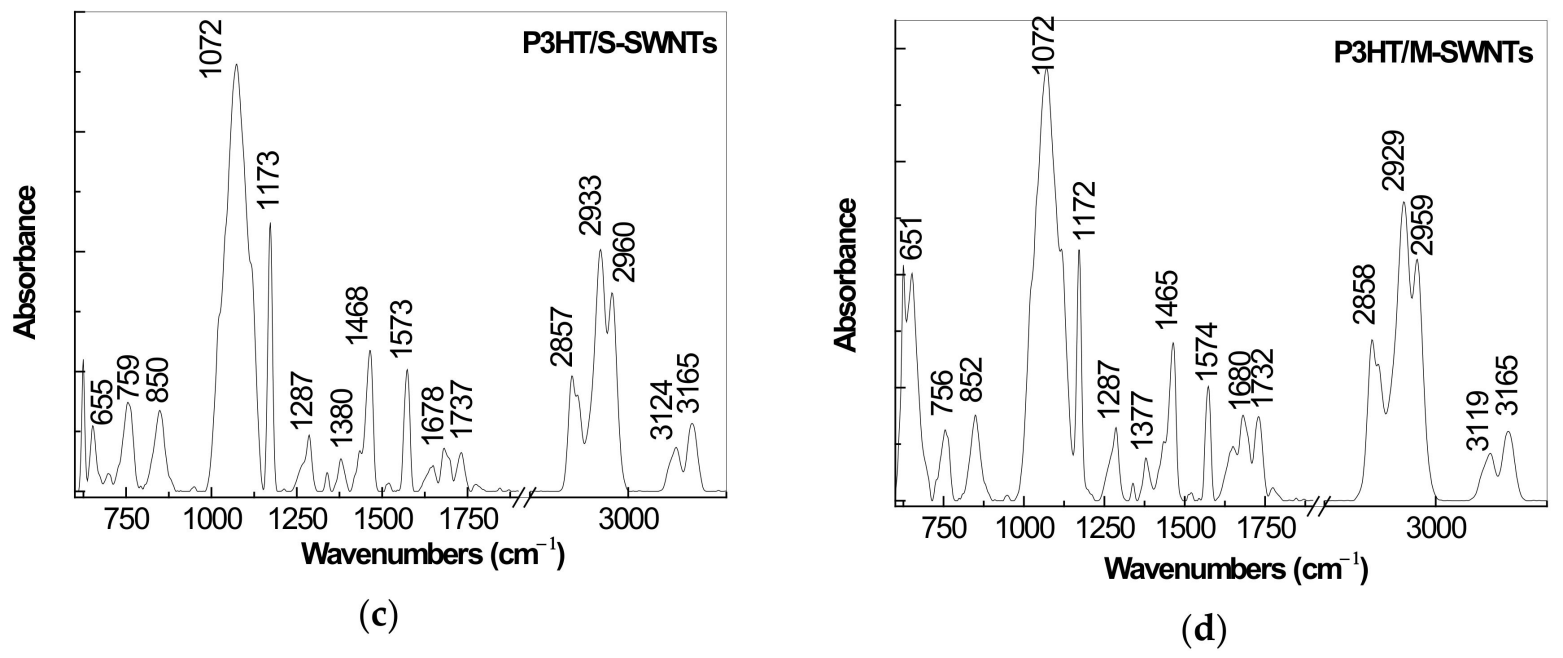
FTIR spectra of P3HT (**a**) and their composites of the type P3HT/M + S-SWNTs (**b**), P3HT/S-SWNTs (**c**) and P3HT/M-SWNTs (**d**), obtained by cyclic voltammetry onto the rough Au electrodes.

In order to prove that on the surface of the gold electrode before and after the modification with M + S-SWNTs, M-SWNTs and S-SWNTs has been generated P3HT, by above mentioned electrochemical process, in Figure 3 are shown the IR spectra in the four cases. Figure 3a highlights the IR bands of the P3HT film, synthesized by electrochemical method, having the maxima at 727, 833, 1076, 1173, 1254, 1377, 1464, 1528, 1574, 1681, 1778, 2858, 2929, 2959 and 3165 cm^−1^. The IR bands at 833, 1377, 1574, 2959 and 3124–3165 cm^−1^ belong to [BMIM]PF_6_, they being assigned to the vibrational modes of PF_6_ asymmetrical stretching, C-C stretching, CH_3_(N) stretching, propyl HCH asymmetrical stretching and ring HCCH asymmetrical stretching, respectively [26]. The presence of these IR bands can be explained as a result of the generation of new π-π* bonds between the C=C bonds of thiophene ring of P3HT and the C=C or C=N bonds of imidazolium ring of [BMIM]PF_6_.

**Scheme 2:**
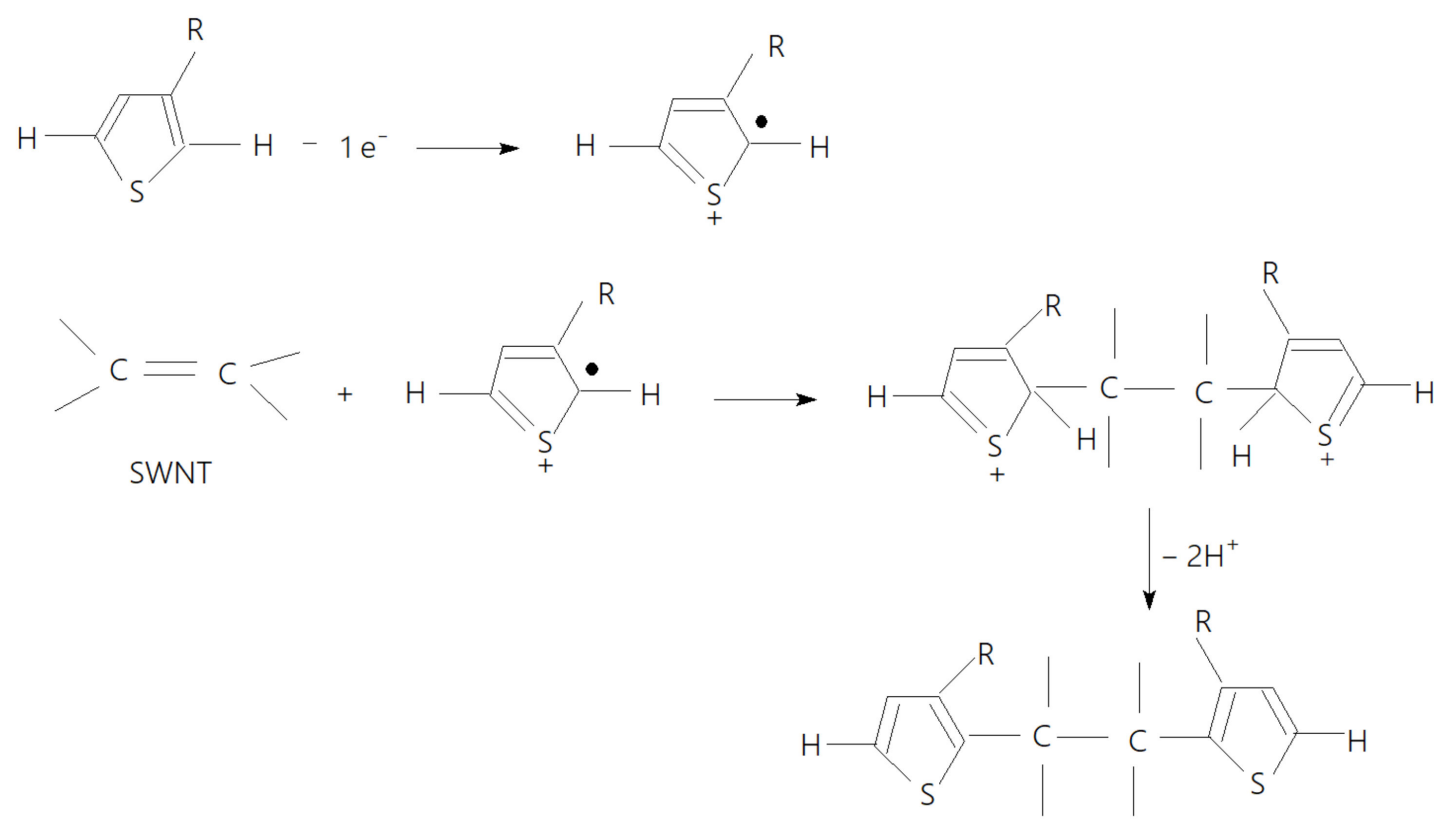

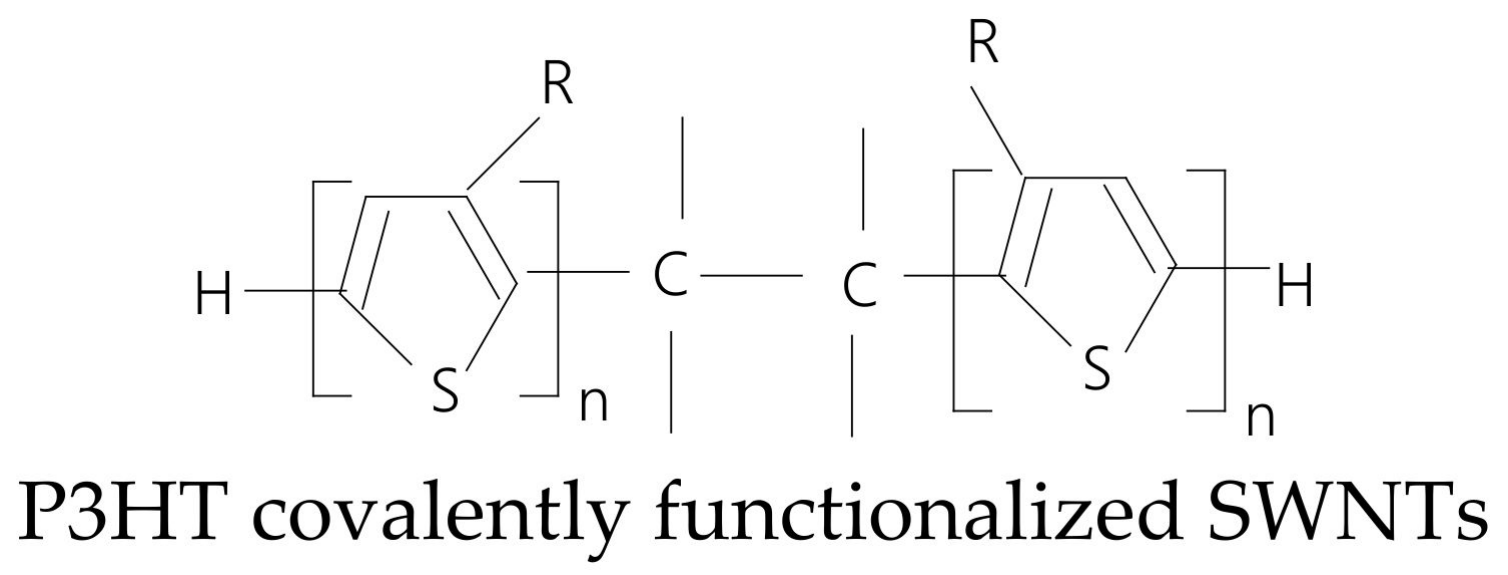
The reaction mechanism of the electrochemical polymerization of 3HT in the presence of CNTs. R corresponds to the hexyl group.

In comparison with the FTIR spectrum of P3HT electrosynthetized onto the Au support (Figure 3a), the following changes are reported in the case of composites based on P3HT and CNTs of the type M + S-SWNTs, S-SWNTs and M-SWNTs: (i) a decrease in the absorbance of the IR band situated in the 1600–1750 cm^−1^ spectral range as remarked in Figure 3b–d; (ii) an increase in the absorbance of the IR band localized in the 3100–3175 cm^−1^ spectral range (Figure 3b–d); and (iii) an increase of the ratio between the absorbances of the IR bands at 1464–1468 and 2930 cm^−1^ from 0.37 (Figure 3a) to 0.48 (Figure 3b), 0.58 (Figure 3c) and 0.52 (Figure 3d). These variations indicate the generation both of π-π* bonds between the C=C bonds from basal plane of graphitic lattice of SWNTs with the C=C bonds of thiophene ring of P3HT, when both a non-covalent functionalization of CNTs with P3HT and a covalent functionalization of CNTs with P3HT occur, the last inducing hindrance steric effects observed by the higher absorbance of the IR bands localized in the 3100–3175 cm^−1^ spectral range, which were assigned to the vibrational mode of the imidazolium ring HCCH asymmetrical stretching of [BMIM]PF_6_, as a consequence of the chemical adsorption of the ionic liquid onto CNTs surface [27]. The electrochemical mechanism which describes the covalently functionalization of CNTs with P3HT is shown in Scheme 2.

Summarizing these results, we can conclude that the chemical synthesis of the composites based on P3HT and CNTs of the type M + S-SWNTs, S-SWNTs and M-SWNTs, involves a non-covalent functionalization of CNTs with P3HT, while the electrochemical synthesis leads both to a non-covalent and covalent functionalization of CNTs with P3HT.

### 2.2. Photoluminescence of the P3HT/CNTs Composites Prepared by Chemical and Electrochemical Methods

**Figure 4:**
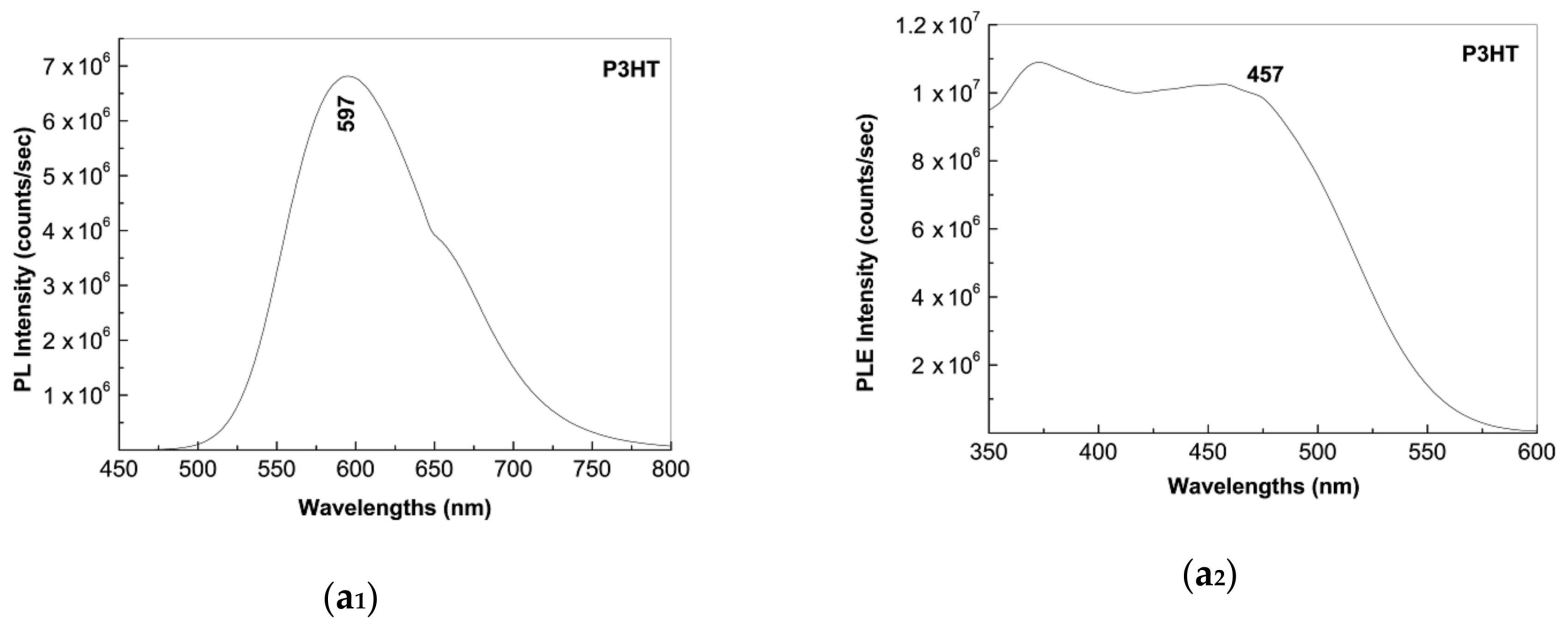

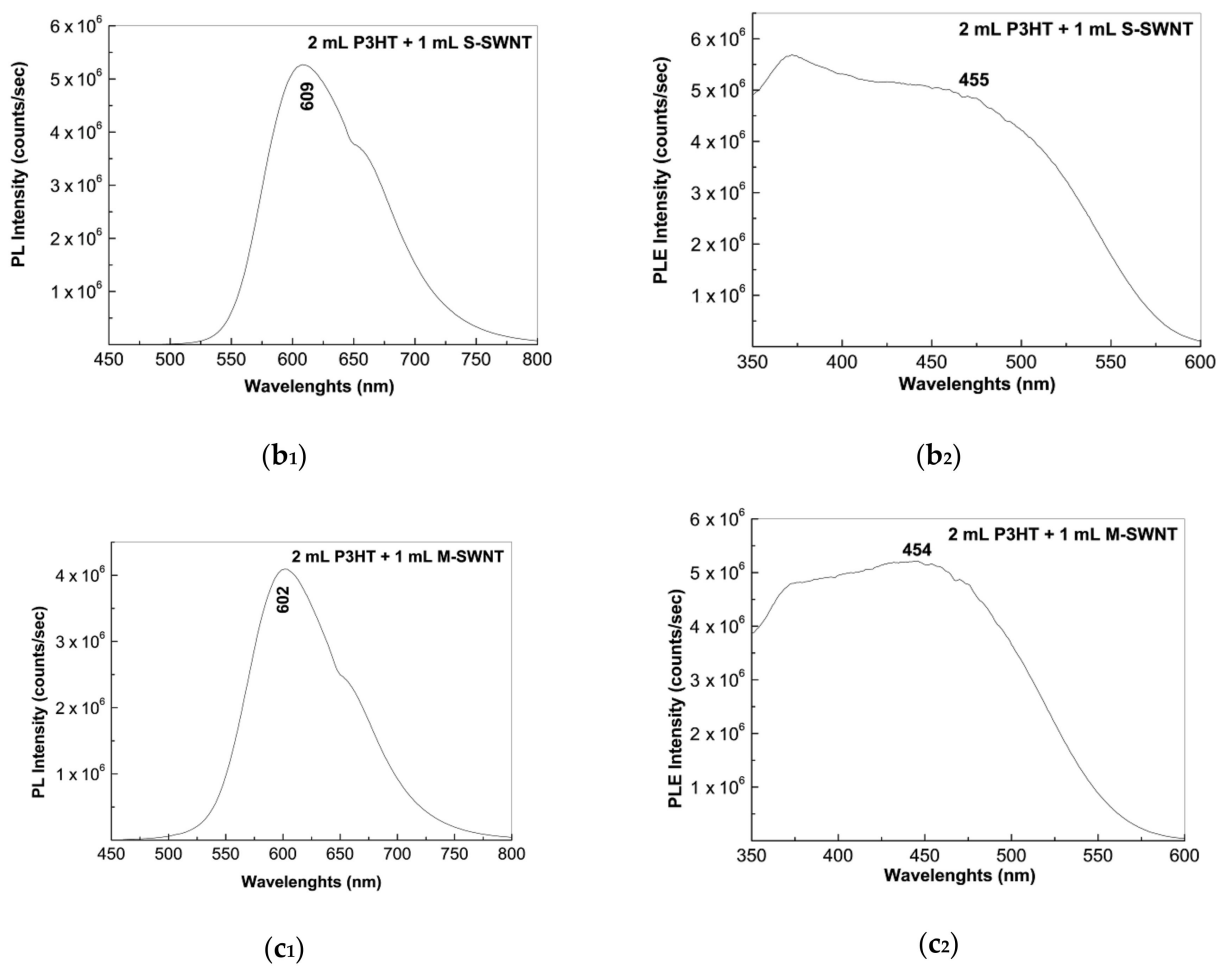
The PL (1) and PLE (2) spectra of the solution of P3HT in DMF:H_2_O before (**a_1_**,**a_2_**) and after the chemical interaction with S-SWNTs (**b_1_**,**b_2_**) and M-SWNTs (**c_1_**,**c_2_**), recorded at the excitation and emission wavelength equal to 440 nm and 620 nm, respectively.

**Figure 5:**
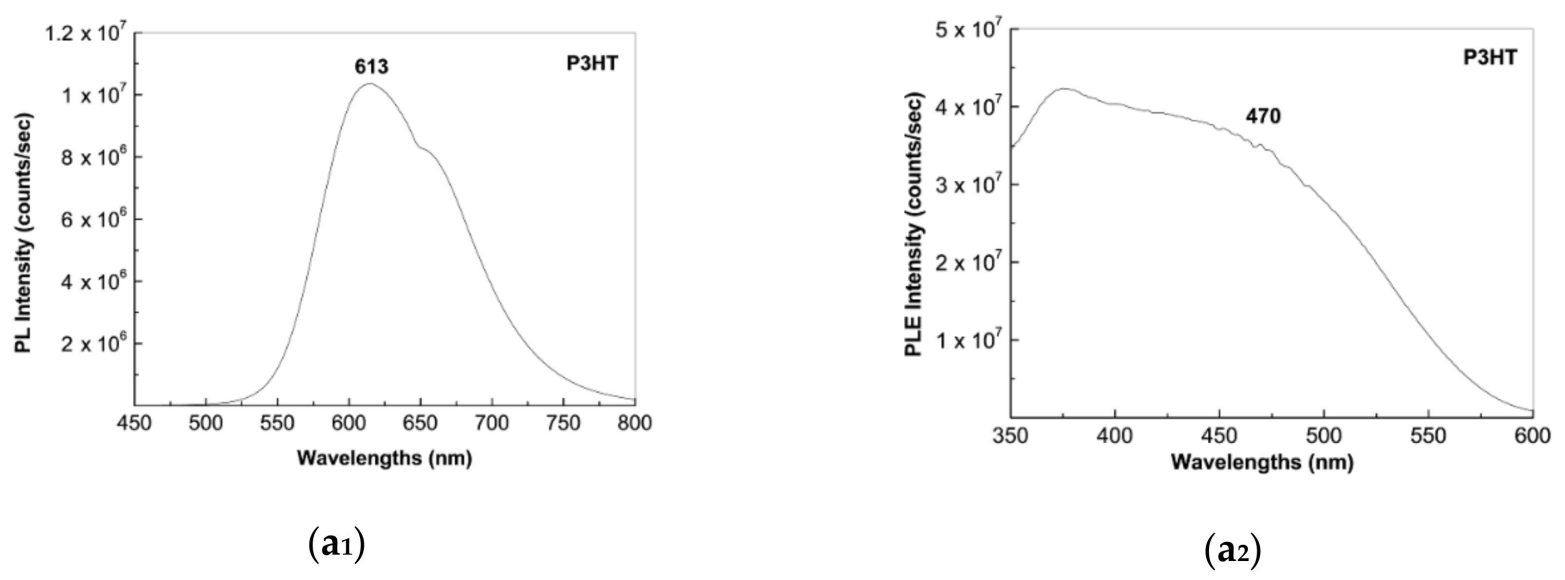

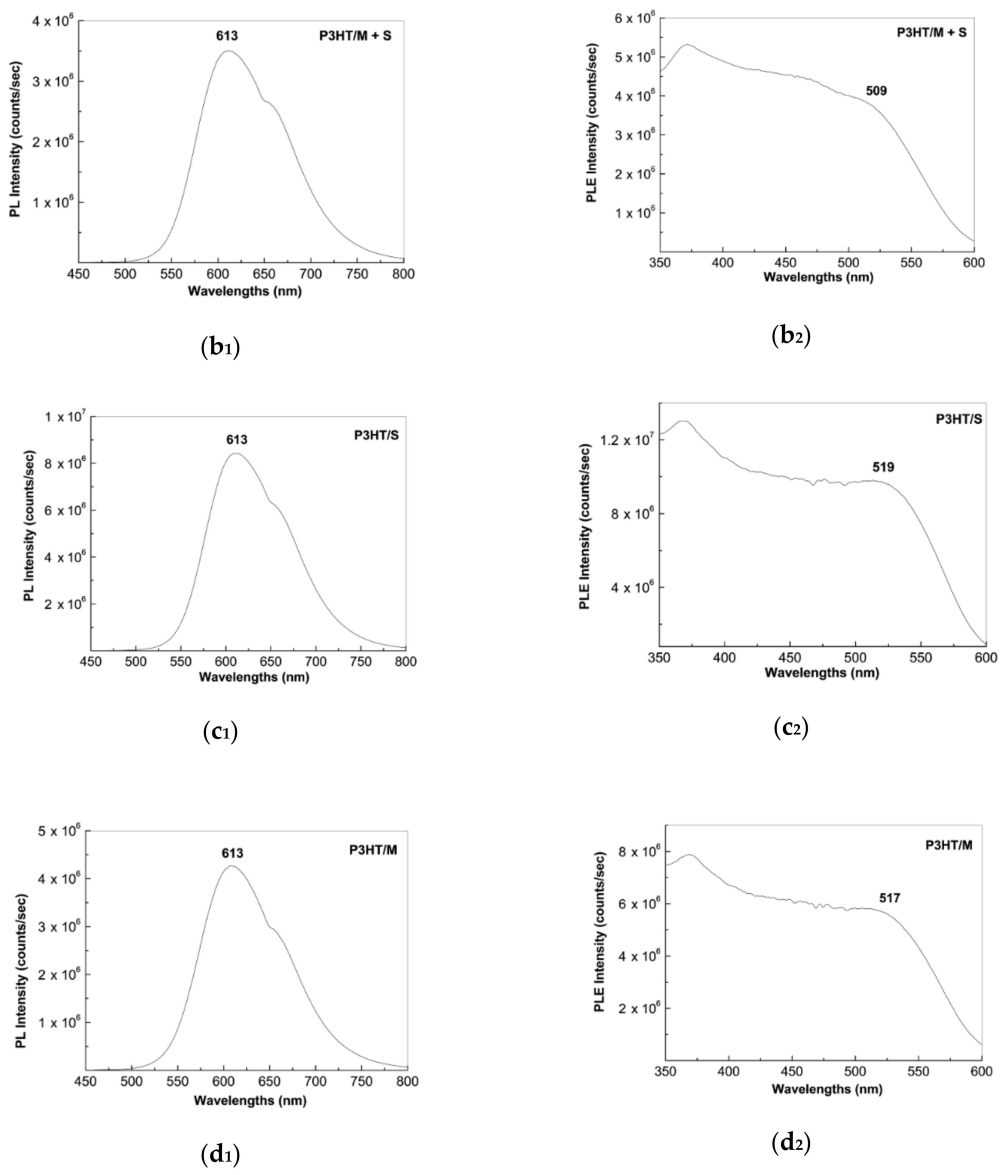
The PL (1) and PLE (2) spectra of the films of P3HT (**a_1_**,**a_2_**) and their composites, obtained by chemical interaction with M + S-SWNTs (**b_1_**,**b_2_**), S-SWNTs (**c_1_**,**c_2_**) and M-SWNTs (**d_1_**,**d_2_**) deposited onto rough Au supports, recorded at the excitation and emission wavelengths of 440 nm and 620 nm, respectively.

Figure 4(a_1_,a_2_) show the PL and PLE spectra of the semi-aqueous solution of P3HT in DMF:H_2_O (volumetric ratio equal to 1:2), recorded at the excitation wavelength of 440 nm and the emission wavelength of 620 nm, respectively. PLE spectrum of P3HT is characterized by a band with the maximum at 457 nm, while the PL spectrum of P3HT is characterized by an emission band with the maximum at 597 nm. According to Figure 4(b_2_,c_2_), the interaction of P3HT with S-SWNTs and M-SWNTs leads to a change in the profile, consisting into a widening of the PLE band to low energies. In addition, the interaction of P3HT with S-SWNTs and M-SWNTs leads to a shift of the PL band of macromolecular compound from 597 nm (Figure 4(a_1_)) to 609 nm (Figure 4(b_1_)) and 602 nm (Figure 4(c_1_)), simultaneous with a change in the intensity of PL band of the polymer from 6.85 × 10^6^ counts/s (Figure 4(a_1)_) to 5.26 × 10^6^ counts/s (Figure 4(b_1_)) and 4.78 × 10^6^ counts/s (Figure 4(c_1_)). A similar behavior is remarked in the case of the films deposited onto rough Au supports, prepared from the solutions of P3HT and CNTs of the type S-SWNTs and M-SWNTs. Figure 5 is relevant in this sense. Figure 5(a_2_,c_2_) and Figure 5(d_2_) highlight a widening of the PLE band to low energies more important in comparison with that reported in Figure 4(a_2_,c_2_). A diminution of the P3HT PL band intensity is remarked to occur from 1.03 × 10^7^ counts/s (Figure 5(a_1_)) to 8.42 × 10^6^ counts/s (Figure 5(c_1_)) and 4.26 × 10^6^ counts/s (Figure 5(d_1_)). This result confirms that lower PL band intensity in the case of the P3HT/M + S-SWNTs composite of 3.49 × 10^6^ counts/s (Figure 5(b_1_)) origins in the presence of the semiconducting and metallic nanotubes, which have the role of the P3HT PL quenching agents.

**Figure 6:**
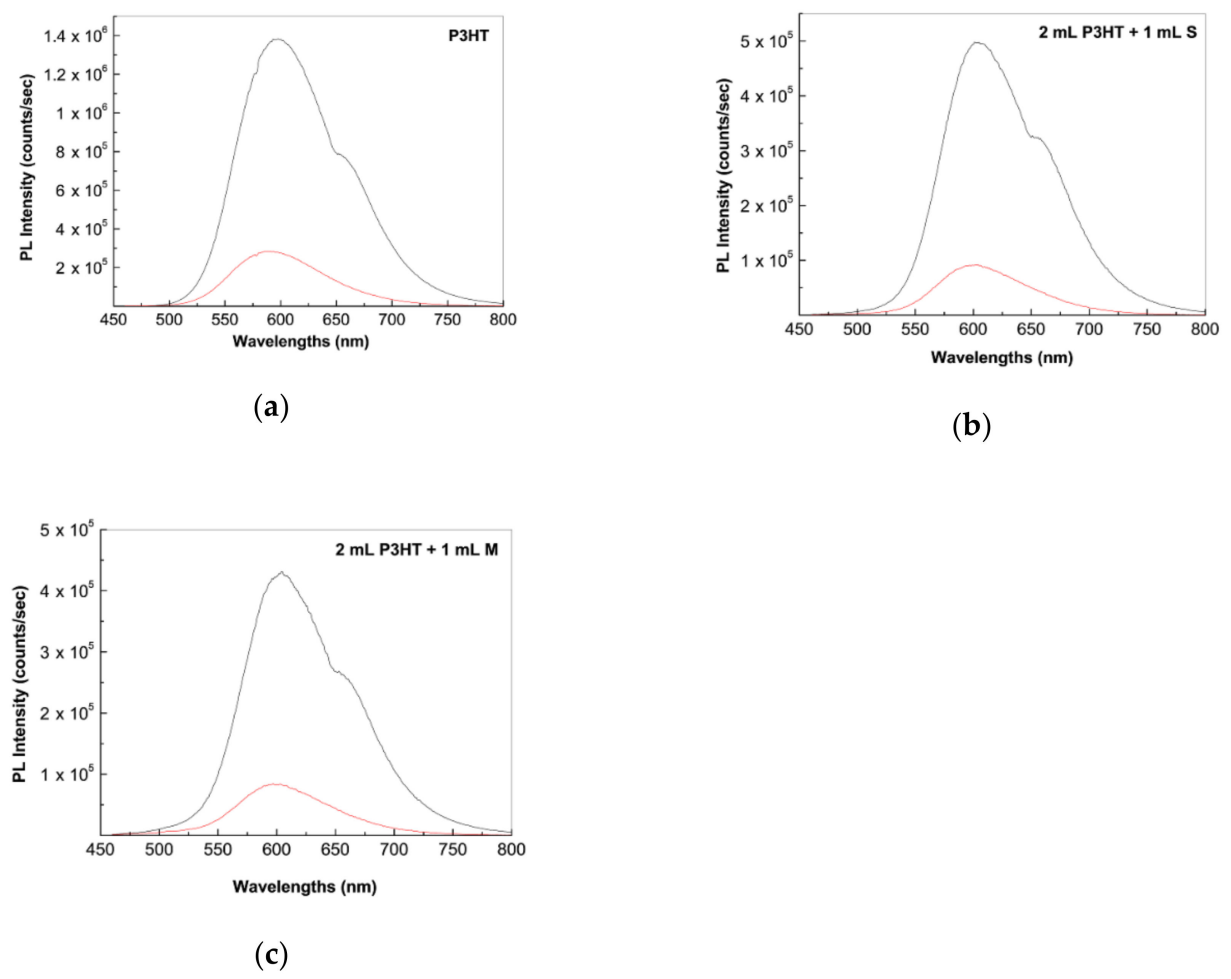
The polarized PL spectra of the solutions of P3HT before (**a**) and after their interaction with S-SWNTs (**b**) and M-SWNTs (**c**). Black and red curves correspond to the PL spectra recorded when the emission and excitation polarizers are both in horizontal and vertical positions.

In order to assess the angle of the binding (*θ*_PL_) of P3HT onto the surface of S-SWNTs and M-SWNTs, when the samples were prepared by chemical interaction of polymer with CNTs, the anisotropic PL measurements were carried out on samples of the type solutions and films deposited onto the rough Au supports. From the PL spectra recorded in polarized light shown in Figure 6 we have calculated the PL anisotropy value with the relation *r* = (*I*_VV_ − *GI*_VH_)/(*I*_VV_ + 2*GI*_VH_), where the calibration factor of the spectrophotometer is noted *G* = *I*_HV_/*I*_HH_, the measured PL intensity when the emission and excitation polarizers are both in vertical and horizontal positions being abbreviated with *I*_VV_ and *I*_HH_ [28]. Thus, the *r* values in the case of the solution of P3HT, P3HT/S-SWNTs and P3HT/M-SWNTs were equal to 0.045, 0.018 and 0.037. The *θ*_PL_ values of P3HT in the absence and in the presence of S-SWNTs and M-SWNTs between the macromolecular compound adsorption and emission dipoles were calculated with the relation *r* = 0.4[(3cos^2^*θ*_PL_ − 1)/2] [28,29], these being equal to 50.2°, 52.9° and 51.2°, respectively.

**Figure 7:**
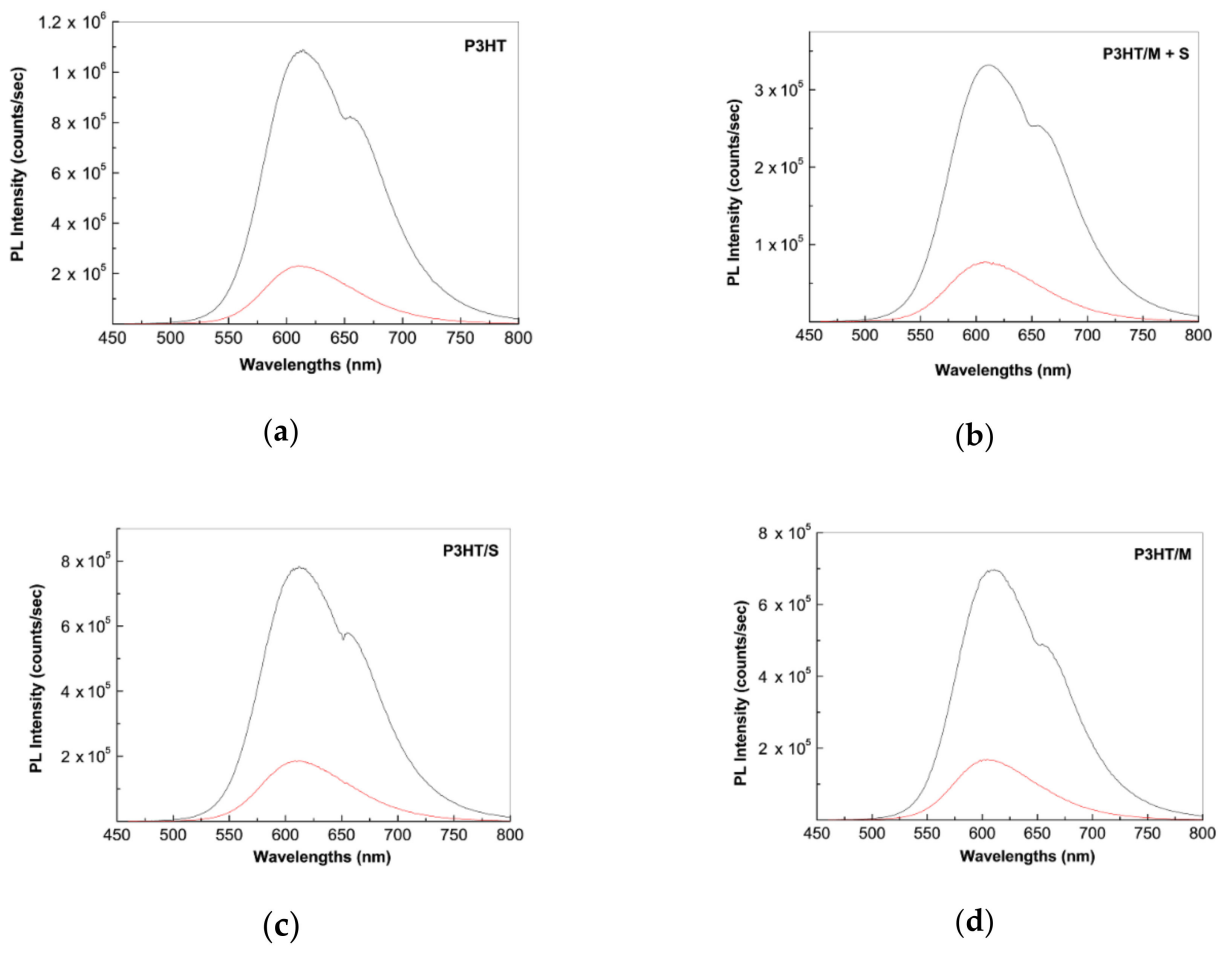
The polarized PL spectra of the P3HT films before (**a**) and after the chemical interaction with M + S-SWNTs (**b**), S-SWNTs (**c**) and M-SWNTs (**d**) and the deposition onto the rough Au supports. Black and red curves correspond to the PL spectra recorded when the emission and excitation polarizers are both in horizontal and vertical positions.

Figure 7 shows the PL spectra recorded in polarized light of the films of P3HT and its composites P3HT/M + S-SWNTs, P3HT/S-SWNTs and P3HT/M-SWNTs. Using above protocol, the PL anisotropy (*r*) value equal to 0.032, 0.003, 0.032 and 0.1071 were calculated in the case of the films of P3HT and its composites P3HT/M + S-SWNTs, P3HT/S-SWNTs and P3HT/M-SWNTs deposited onto rough Au supports. The *θ*_PL_ values of P3HT and its composites P3HT/M + S-SWNTs, P3HT/S-SWNTs and P3HT/M-SWNTs deposited onto rough Au supports were equal to 51.4°, 54.4°, 51.6° and 52.9°, respectively. These results indicate that by the chemical interaction of P3HT with CNTs of the type M + S-SWNTs, S-SWNTs and M-SWNTs, that is, when results composites of the type CP non-covalently functionalized CNTs, only a small variation in the angle of the binding of macromolecular compound onto CNTs surface takes place.

**Figure 8:**
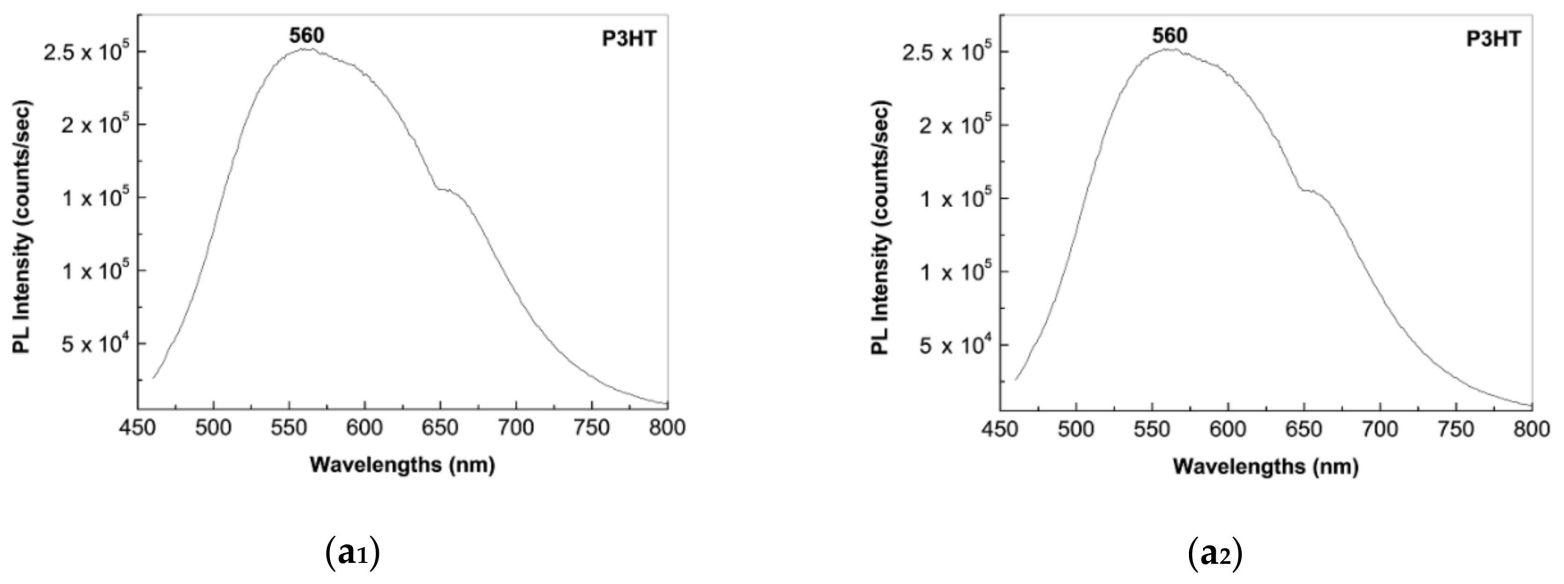

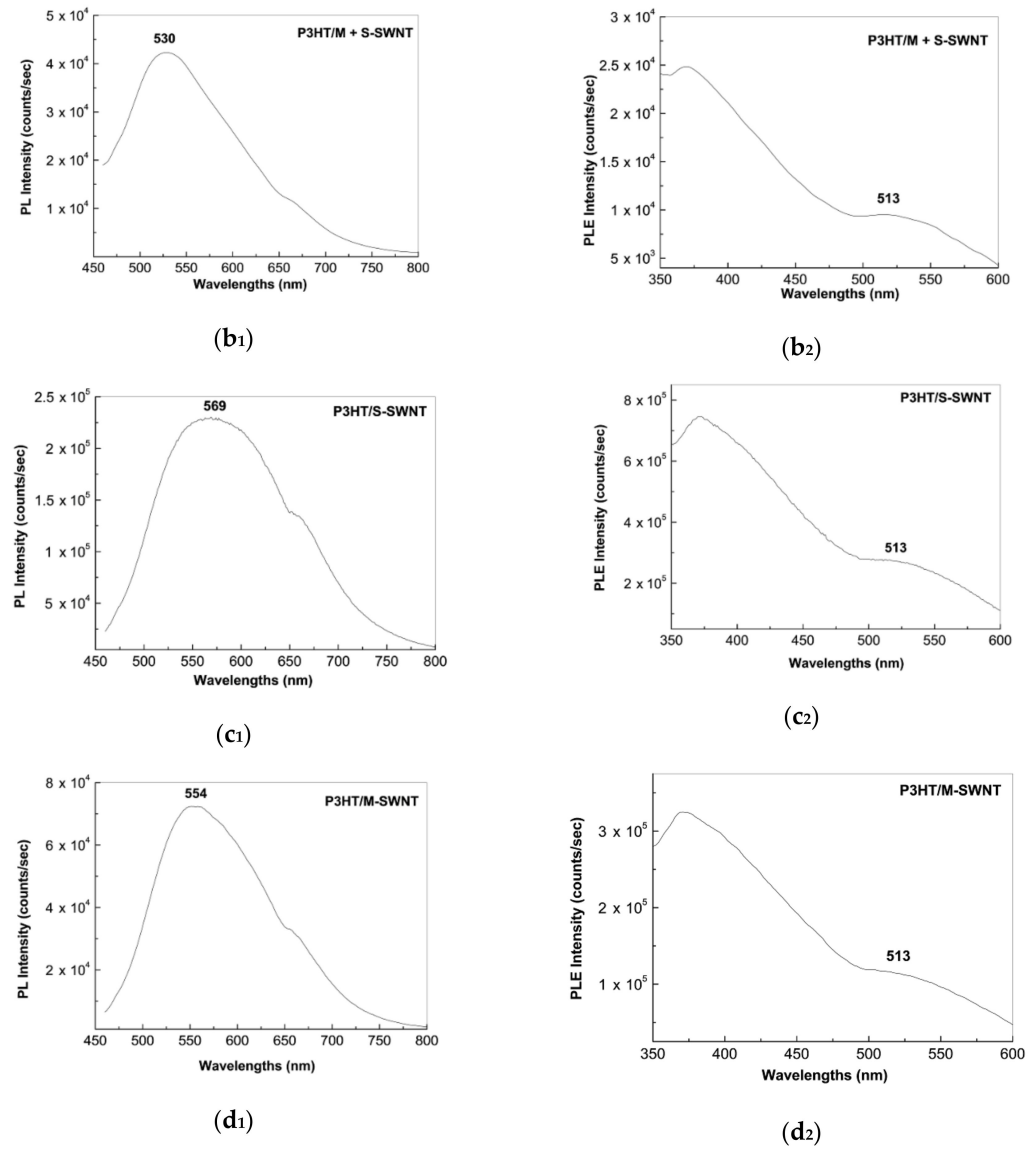
The PL (1) and PLE (2) spectra of the films of P3HT (**a_1_**,**a_2_**) and their composites, with M + S-SWNTs (**b_1_**,**b_2_**), S-SWNTs (**c_1_**,**c_2_**) and M-SWNTs (**d_1_**,**d_2_**) obtained by the electrochemical polymerization of 3HT in the presence of [BMIM]PF_6_ onto rough Au supports. The PL and PLE spectra were recorded at the excitation and emission wavelengths of 440 nm and 620 nm, respectively.

**Figure 9:**
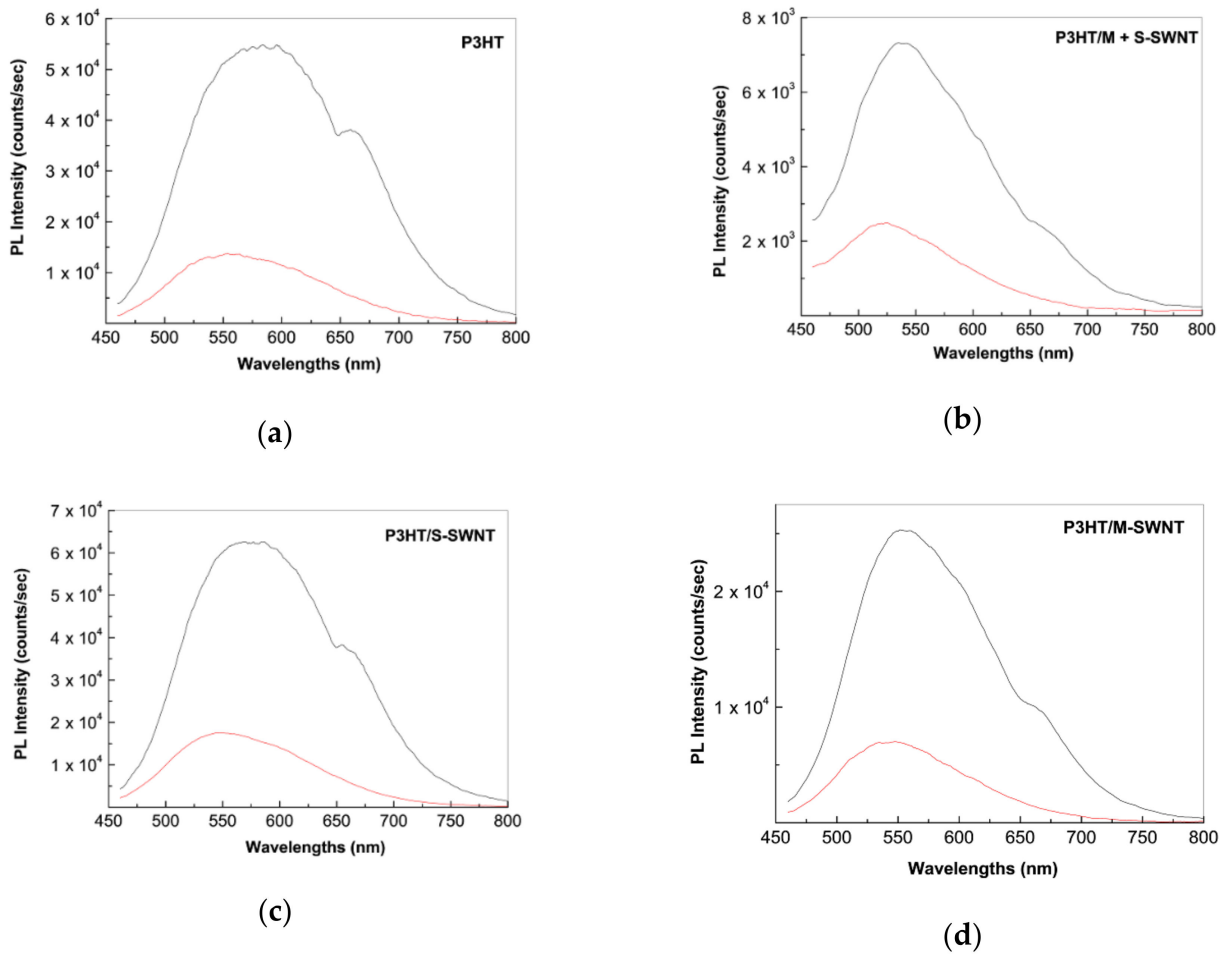
The polarized PL spectra of the P3HT in the initial state (**a**) and their composites with M + S-SWNTs (**b**), S-SWNTs (**c**) and M-SWNTs (**d**), prepared by the electrochemical polymerization of 3HT in the presence of [BMIM]PF_6_ onto rough Au supports. Black and red curves correspond to the PL spectra recorded when the emission and excitation polarizers are both in horizontal and vertical positions.

Figure 8 and Figure 9 show the PL properties of the composites based on P3HT and CNTs of the type M + S-SWNTs, S-SWNTs and M-SWNTs prepared by cyclic voltammetry.

The recording of the same cyclic voltammograms number onto the rough Au electrode before and after the modification with CNTs of the type M + S-SWNTs, S-SWNTs and M-SWNTs induces in Figure 8: (i) a shift of the PLE band maximum of P3HT from 531 nm (Figure 8(a_2_)) to 513 nm in the case of the composites P3HT/M + S-SWNTs (Figure 8(b_2_)), P3HT/S-SWNTs (Figure 8(c_2_)) and P3HT/M-SWNTs (Figure 8(d_2_)), (ii) a change in the emission band maximum from 560 nm (Figure 8(a_1_)) to 530 nm (Figure 8(b_1_)), 569 nm (Figure 8(c_1_)) and 554 nm (Figure 8(d_1_)) and (iii) a variation in the intensity of the PL band from 2.51 × 10^5^ counts/s (Figure 8(a_1_)) to 4.22 × 10^4^ counts/s (Figure 8(b_1_)), 2.3 × 10^5^ counts/s (Figure 8(c_1_)) and 7.24 × 10^4^ counts/s (Figure 8(d_1_)). These results clearly demonstrate that the semiconducting and metallic CNTs have the role of P3HT PL quenching agents.

The PL spectra in polarized light of the samples prepared by cyclic voltammetry are shown in Figure 9. Using above protocol, the following values for r and *θ*_PL_ are calculated in the case: (i) P3HT, 0.264 and 28.5°; (ii) P3HT/M + S-SWNTs, 0.383 and 9.6°; (iii) P3HT/S-SWNTs, 0.337 and 19° and (iv) P3HT/M-SWNTs, 0.544 and 0°. These values reported in the case of the samples prepared by electrochemical method are lower in contrast with those obtained by chemical interaction of the two constituents.

In our opinion, this fact origins in the functionalization process of CNTs in the case of the samples prepared by electrochemical and chemical method, that is, when P3HT covalently functionalized CNTs and P3HT non-covalently functionalized CNTs, respectively, are obtained. The change of the *θ*_PL_ value in the case of the P3HT/M + S-SWNTs composite, in comparison with P3HT, origins metallic nanotubes. The *θ*_PL_ value equal to 0° indicates that in the case of the PHT/M-SWNTs composite, the excitation and emission transition dipoles of the polymer are parallel to the basal plane of graphitic lattice of CNTs.

### 2.3. The SERS and UV-VIS-NIR Spectra of the P3HT/CNTs Composites

**Figure 10:**
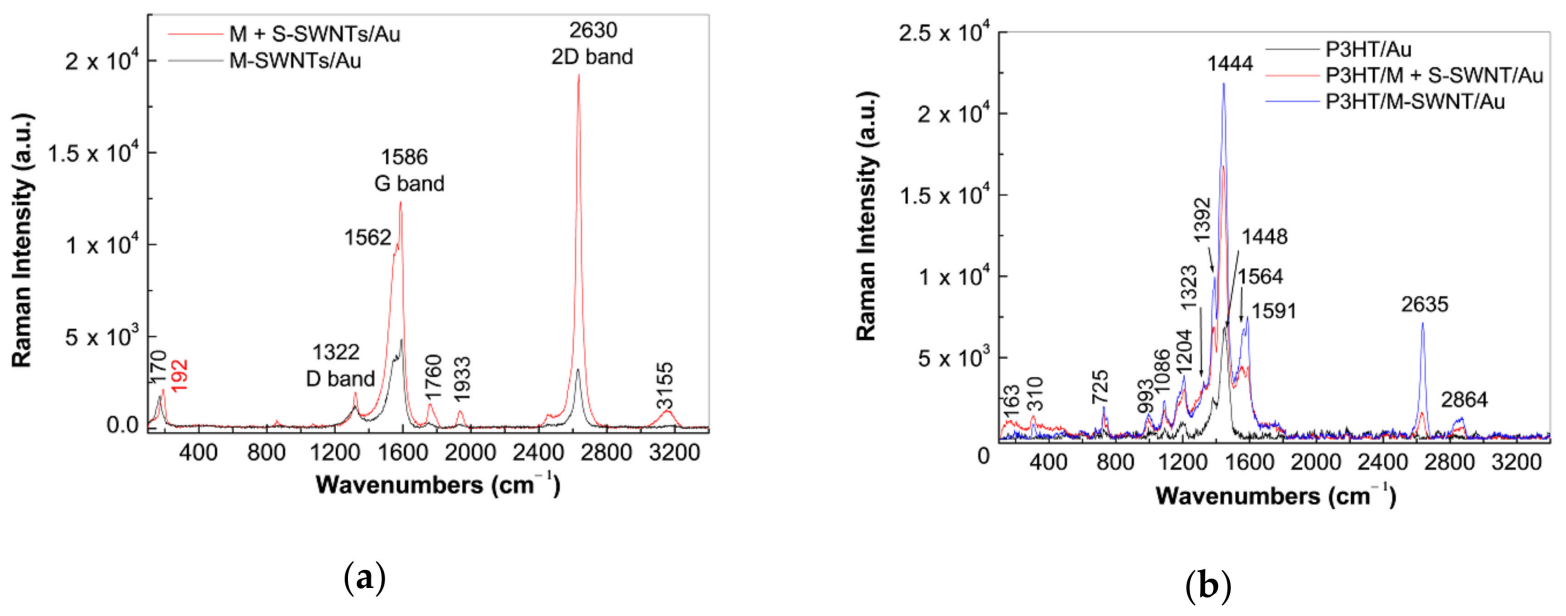
Surface-enhanced Raman scattering (SERS) spectra of: (**a**) M + S–SWNTs (black curve) and M-SWNTs (red curve) and (**b**) P3HT (black curve) and their composites with M + S-SWNTs/P3HT (red curve) and M-SWNTs (blue curve), obtained by the chemical interaction. All SERS spectra were recorded at the excitation wavelength of 633 nm.

**Figure 11:**
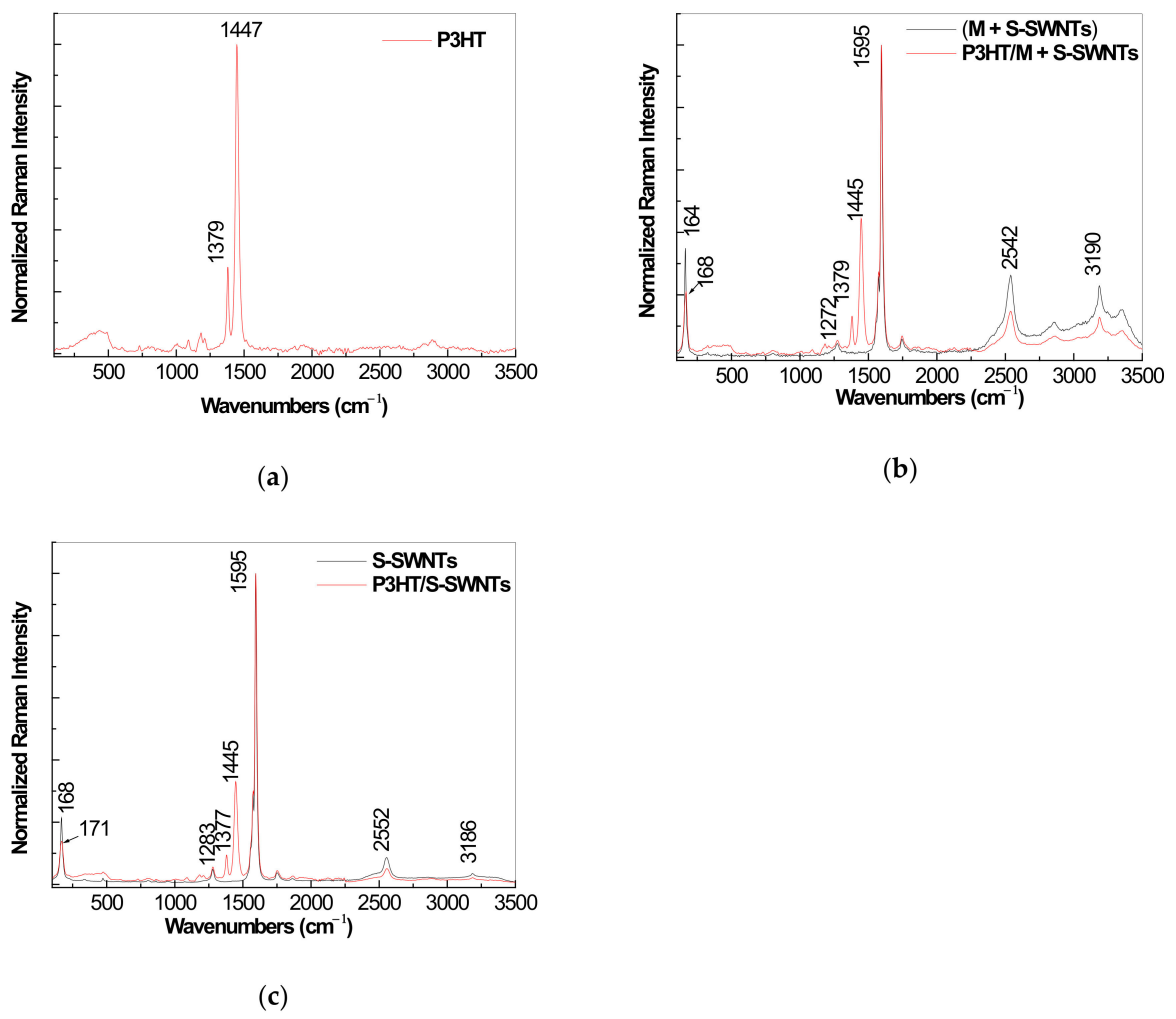
SERS spectra of: (**a**) M + S-SWNTs (black curve) and S-SWNTs (blue curve) and (**b**) P3HT (black curve) and their composites with M + S-SWNTs/P3HT (red) and S-SWNTs/P3HT (**c**), obtained by the chemical interaction. All SERS spectra were recorded at the excitation wavelength of 1060 nm.

**Figure 12:**
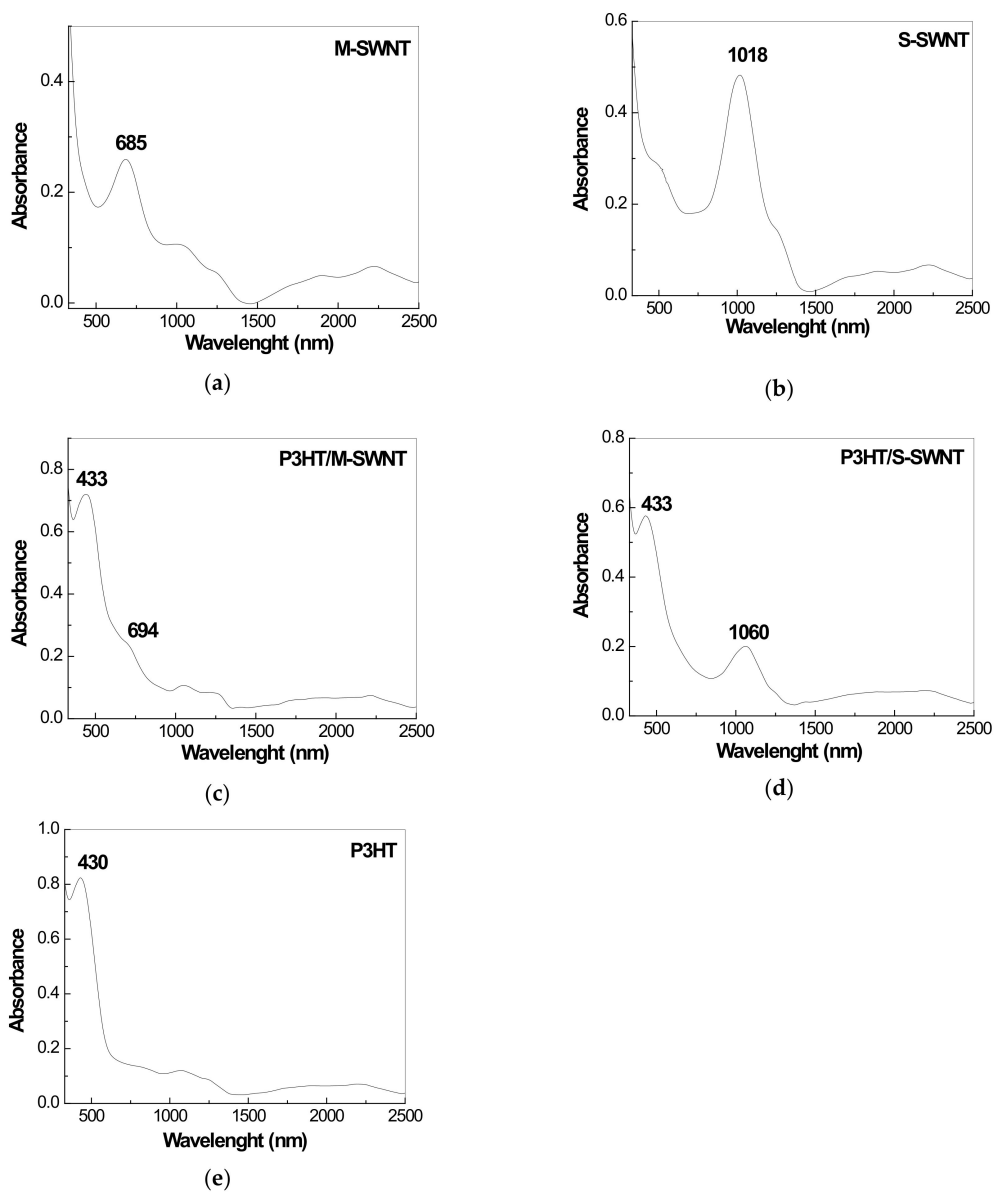
UV-VIS-NIR spectra of: M-SWNT (**a**), S-SWNTs (**b**) and their composites with P3HT, obtained by the chemical interaction of the two constituents, that is, P3HT/M-SWNT (**c**), P3HT/S-SWNT (**d**) and P3HT (**e**).

Figure 10, Figure 11 and Figure 12 show some information on the SERS and UV-VIS-NIR spectra of composite materials prepared by the chemical interaction of the two constituents, namely P3HT and CNTs of the type M-SWNTs and S-SWNTs. Figure 10a shows the SERS spectra of M + S-SWNTs (black curve) and M-SWNT (red curve). Regardless of CNTs, three spectral ranges are shown in Figure 10, when Raman spectra are recorded at the excitation wavelength of 633 nm, that is, in resonance conditions for metallic SWNTs: (i) the low frequencies range between 100–250 cm^−1^ assigned to the radial breathing mode (RBM), the maximum of the Raman line of this vibrational mode is peaked at 170 and 192 cm^−1^ in the case of M + S-SWNTs and M-SWNTs, respectively; (ii) the 1000–1750 cm^−1^ spectral range, where is observed an intense Raman band with the maximum at 1586 cm^−1^ assigned to the tangential mode (labeled as G band), which is accompanied of a component at 1562 cm^−1^ known as Breit-Wigner-Fano component that induces an asymmetrical profile of the G band, this being due to the electron-phonon interaction; in this spectral range, a low intensity Raman band with the maximum at 1322 cm^−1^ was assigned to defects or the disorder state induced in the graphitic lattice of SWNTs; and (iii) high frequencies spectral range between 2400–3500 cm^−1^, where the Raman lines have the maxima at twice the D and G band frequency [30]. Knowing the frequency of the RBM Raman line of SWNTs and using the relation *ν* (cm^−1^) = 248/d (nm) [30], the diameter of nanotubes can be calculated. Thus, the diameter of M + S-SWNTs and M-SWNTs was calculated to be 1.47 nm and 1.25 nm, respectively.

The SERS spectrum of the P3HT films (black curve in Figure 10b) is dominated of an intense line with the maximum at 1448 cm^−1^, these being accompanied of other Raman lines of low intensity peaked at 725, 993, 1086, 1204 and 1383 cm^−1^. The Raman lines peaked at 725, 993, 1086, 1204, 1383 and 1448 cm^−1^ are assigned to the vibrational modes C_α_-S-C_α’_ deformation, C_β_-C_alkyl_ stretching, C_β_-H bending, C_α_-C_α’_ stretching + C_β_-H bending, C_β_-C_β’_ stretching and C_α_ = C_β_ stretching [31]. In comparison with the SERS spectra of the two constituents of the P3HT/M + S-SWNTs and P3HT/M-SWNTs composites, the following differences are remarked in Figure 10b: (i) a down-shift of the Raman line of P3HT from 1448 cm^−1^ to 1444 cm^−1^; (ii) an up-shift of the Raman lines of SWNTs from 1586 and 2630 cm^−1^ to 1591 and 2635 cm^−1^, simultaneous with a decrease in the intensity of the 2D band; and (iii) a significant decrease in the intensity of the Raman line assigned to RBM simultaneous with a down-shift at 163 cm^−1^. The decrease in the intensity of the 2D band was reported to be a signature for an increased number of defects in the SWNTs structure embedded in the polymer matrix [32]. The up-shift of the G band of SWNTs was reported to have origin in the deformation of the C-C bonds of the CNTs [33], which in the presence case was induced of the π-π stacking interactions between the two constituents.

Figure 11a shows the SERS spectrum of the P3HT film deposited onto rough Au support, recorded at the excitation wavelength of 1064 nm. As main feature of the SERS spectrum of the P3HT film at the excitation wavelength of 1064 nm (Figure 11a) we note the intense Raman line with the maximum at 1447 cm^−1^, which is accompanied of a Raman line of low intensity at 1379 cm^−1^. Black curves in Figure 11b,c show SERS spectra of the M + S-SWNTs and S-SWNTs, which are characterized by: (i) the Raman lines of RBM with maxima at 164 and 168 cm^−1^, respectively; (ii) an intense G Raman line with the maximum at 1595 cm^−1^ which is accompanied of a D band having the maximum at 1272 cm^−1^ and 1283 cm^−1^, respectively; and (iii) two order of the D and G bands peaked at 2542 cm^−1^ or 2552 cm^−1^ and 3190 cm^−1^ or 3186 cm^−1^, respectively, in the case of M + S-SWNTs and S-SWNTs. According to Figure 11b,c, the follows changes are remarked in the Raman spectra of the two constituents of the P3HT/M + S-SWNTs and P3HT/S-SWNTs composites: (i) an up-shift of the Raman line assigned to RBM from 164 cm^−1^ (black curve in Figure 11b) and 168 cm^−1^ (black curve in Figure 11c) to 168 cm^−1^ (red curve in Figure 11b) and 171 cm^−1^ (red curve in Figure 11c); (ii) the appearance of the Raman line of P3HT at 1379–1377 cm^−1^ and 1445 cm^−1^; and (iii) a decrease in the intensity of the Raman lines localized in the higher frequencies spectral range 2500–3500 cm^−1^. Often, the up-shift of the RBM band was invoked as an evidence for the wrapping of the SWNTs surface with the macromolecular compounds [34] or water molecules [35], when π-π* or hydrogen bonds were established between these constituents.

**Figure 13:**
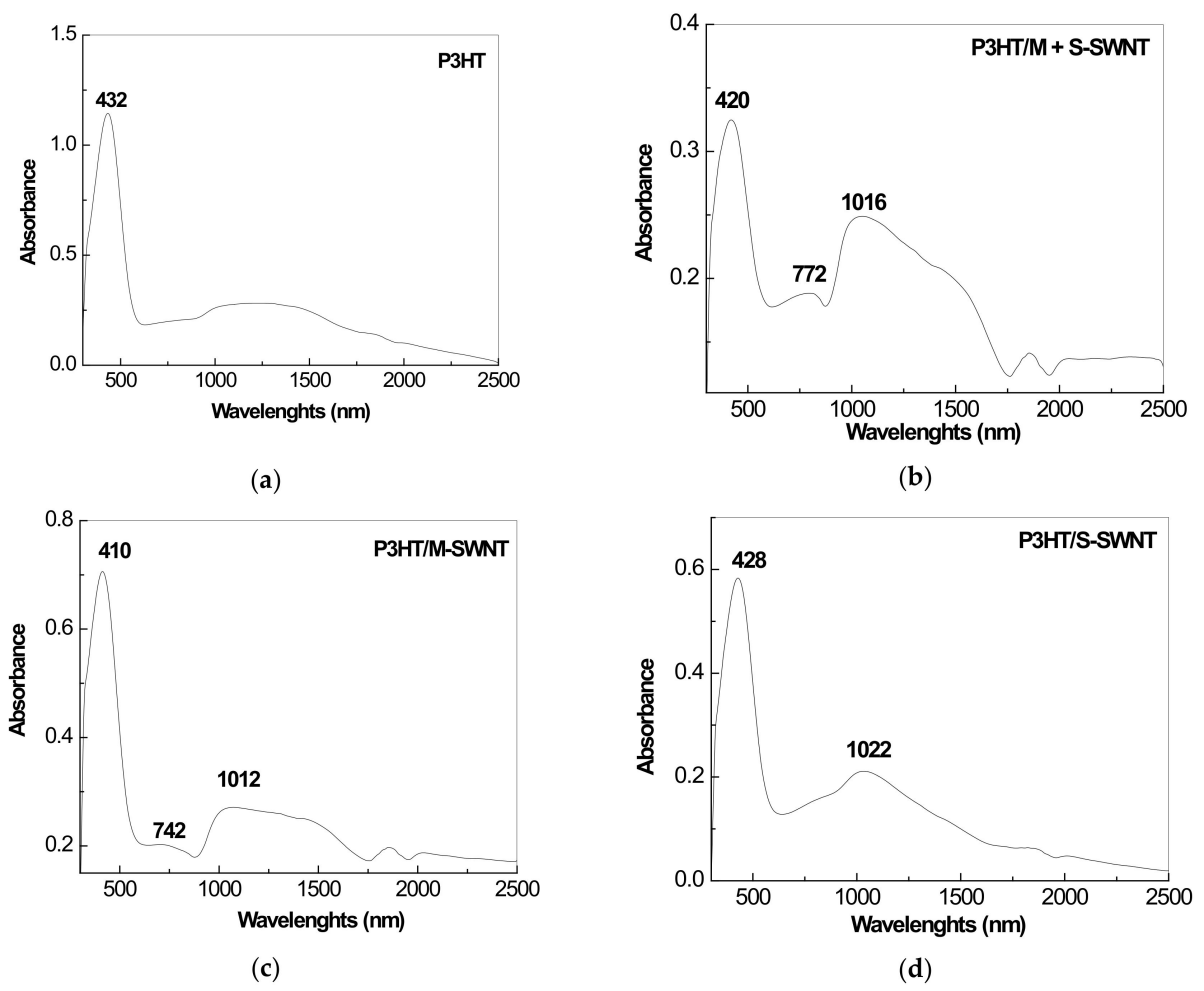
UV-VIS-NIR spectra of the films of: P3HT (**a**) and their composites obtained by the electrochemical polymerization of 3HT in the presence of the CNTs, that is, P3HT/M + S-SWNT (**b**), P3HT/M-SWNT (**c**) and P3HT/S-SWNT (**d**).

Figure 12 shows UV-VIS-NIR spectra of P3HT, M-SWNTs, S-SWNTs and their composites obtained by the chemical interaction of the macromolecular compound and CNTs. The UV-VIS-NIR spectra of the solutions of M-SWNTs, S-SWNTs and P3HT highlight the bands with high absorbance at 685 nm (1.81 eV), 1018 nm (1.22 eV) and 430 nm (2.88 eV), in which the first two were assigned to the M_11_ and S_22_ electronic transitions [23] and the last one was attributed to π-π* transition of P3HT [36,37]. In the case of the P3HT/M-SWNTs and P3HT/S-SWNTs composites, an up-shift of the bands assigned to the M_11_, S_22_ and π-π* transitions from 685 nm (Figure 12a), 1018 nm (Figure 12b) and 430 nm (Figure 12e) to 694 nm (Figure 12c), 1060 nm (Figure 12d) and 433 nm (Figure 12c,d), respectively. These variations originate in the non-covalent functionalization of SWNTs with P3HT. In contrast with the non-covalent functionalization of SWNTs with P3HT, the main difference in the UV-VIS-NIR spectra of P3HT electrochemical synthesized in the presence of M + S-SWNTs, M-SWNTs and S-SWNTs consists in the shift of the band assigned to the π-π* electronic transition from 432 nm (Figure 13a) to 420 nm (Figure 13b), 410 nm (Figure 13c) and 428 nm (Figure 13d).

Similar to previous studies performed on CNTs and other macromolecular compounds [38], in our case the presence of the van Hove singularities of SWNTs in the UV-VIS-NIR spectra of the P3HT/M-SWNT and P3HT/S-SWNT composites indicates that a bundling process of CNTs not occurs, the polymer being deposited onto the surface of individual tubes.

### 2.4. The Mechanism of P3HT PL Quenching Process

**Figure 14:**
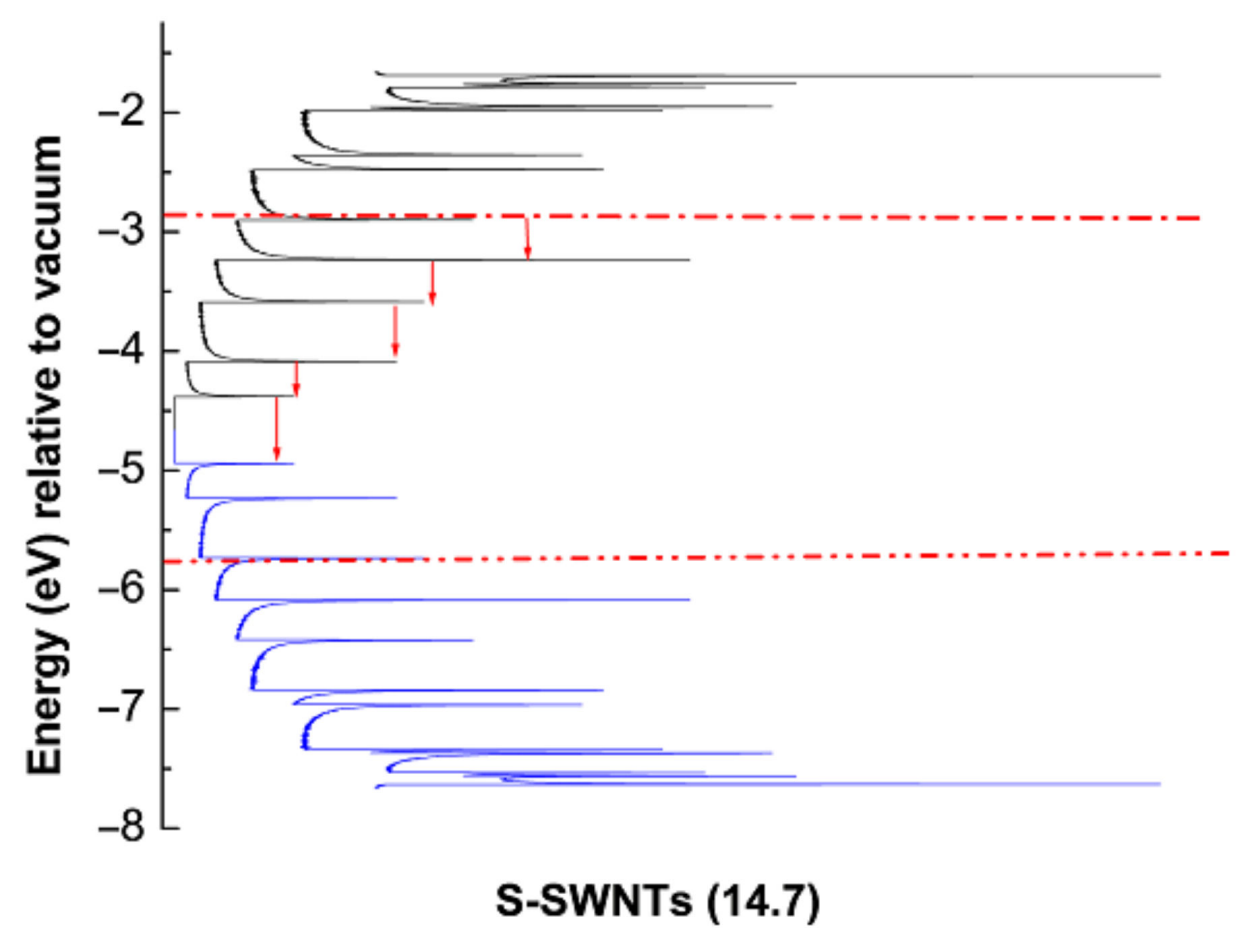
The electronic energy levels diagram of SSWNTs (black and blue solid lines) and P3HT (red dotted lines).

In order to exemplify the potential de-excitation pathways of the P3HT/M-SWNTs and P3HT/S-SWNTs composites in the following we will focus the attention on the energy levels diagram for the particular case of samples obtained by chemical interaction. Figure 14 shows the energy levels diagram of S-SWNTs and P3HT, calculated using the protocol reported in the Ref. [11].

In the case of P3HT, we have calculated the optical band gap to be 2.88 eV, the highest occupied molecular orbital (HOMO) equal to −5.7 eV and the lowest unoccupied molecular orbital (LUMO) situated at −2.82 eV. The HOMO and LUMO levels of the S-SWNTs were determined considering the diameter of S-SWNTs equal to 1.47 nm, which correspond to the chirality (14,7), respectively. Using the work function of −4.66 eV [39], the HOMO/LUMO levels of S-SWNTs are situated at −4.94/−4.38 eV versus the local vacuum level.

The de-excitation pathways take into account for the P3HT/S-SWNTs composite consider that under optical excitation, the exciton which appears onto the P3HT chain is dissociated into an electron and a hole. The electron will be collected by the LUMO level of SWNTs from the proximity and by an internal conversion process this will be passed successively to lower energy LUMO level. In the case of the P3HT/S-SWNTs composites, the electron from the lower energy LUMO level of S-SWNTs will be recombined with the hole from the HOMO level of S-SWNTs (−4.94 eV), which is situated at higher energy in comparison with the HOMO level of P3HT (−5.7 eV).

Regardless of the synthesis method of the P3HT/SWNT composites, that is, chemical or electrochemical, the S_22_ band is clearer observed in the UV-VIS-NIR spectra shown in Figure 12 and Figure 13, in contrast with the M_11_ band. This fact suggests an energy transfer in the P3HT wrapped M-SWNTs, which reduces the polymer PL intensity, similar to that reported in Ref. [40].

## 3. Materials and Methods

P3HT, 3HT, M + S-SWNTs, dimethylformamide (DMF), toluene, sodium dodecyl sulfate (SDS) and 1-butyl-3-methylimidazolium hexafluoro phosphate ([BMIM]PF_6_) were purchased from Sigma-Aldrich company (St. Louis, MO, USA). M-SWNTs (98%) and S-SWNTs (99%) as solutions with the concentration <0.01 wt.% and thick films were bought from NanoIntegris (Quebec, QC, Canada).

In order to obtain the P3HT/CNTs composites by chemical interactions of the two constituents, a solution of 1 mL P3HT 0.1 mg mL^−1^ in DMF was interacted with 2 mL aqueous solution of M-SWNTs or S-SWNTs, all with the concentration <0.01 wt.%, time of 5 min. under ultrasonication. These solutions were used for the studies of the UV-VIS-NIR spectroscopy and PL. In the case of the P3HT/M + S-SWNTs composites, the chemical interaction of P3HT with the M + S-SWNTs solution prepared by the ultrasonication of 10 mg in SDS (20 mL, 32 mM) was carried out [41]. In the case of the studies of PL, FTIR spectroscopy and SERS, the films of P3HT and its composites with CNTs were prepared by the drop casting method when 0.1 mL solutions of P3HT/M + S-SWNTs, P3HT/M-SWNTs or P3HT/S-SWNTs were deposited onto the rough Au supports and then evaporated under vacuum time of 24 h.

A method of preparing P3HT/CNTs composite films was cyclic voltammetry, by the electrochemical polymerization of the aqueous solution of 3HT (0.1 M) in the presence of 5 mL of [BMIM]PF_6_ in the potential range (0; +1.8) V onto the surface of the working electrodes from gold, the auxiliary and reference electrodes being from gold and silver, respectively. The gold working electrode was modified with M + S-SWNTs, S-SWNTs and M-SWNTs using in the case of each electrode a suspension of CNTs prepared by the ultrasonication of the thick films of M-SWNTs and S-SWNTs or of the powder of M + S-SWNTs in toluene, which had a concentration of 0.001g/mL. All cyclic voltammograms were stopped at the potential of 0 V vs. Ag.

UV-VIS-NIR spectra of CNTs and the P3HT/CNTs composites were recorded with an UV-VIS-NIR spectrophotometer, Lambda 950 model, from Perkin Elmer ((PerkinElmer, Inc., Waltham, MA, USA). For the films of P3HT and its composites with CNTs, prepared by chemical and electrochemical methods, quartz supports and ITO electrodes, respectively, were used.

IR spectra of P3HT and their composites with M + S-SWNTs, S-SWNTs and M-SWNTs were recorded with a FTIR spectrophotometer, Vertex 80 model, from Bruker ((Billerica, MA, USA), in the attenuated total reflection (ATR) geometry.

Raman spectra of M + S-SWNTs, S-SWNTs and their composites with P3HT, that is, P3HT/M + S-SWNTs and P3HT/S-SWNTs, were recorded at the excitation wavelength equal to 1064 nm with a FT Raman spectrophotometer RFS100S from Bruker.

Raman spectra of M + S-SWNTs, M-SWNTs and their composites with P3HT, that is, P3HT/M + S-SWNTs and P3HT/M-SWNTs, were recorded at the excitation wavelength equal to 633 nm with a Raman “Confotec CARS” microspectrometer (SOL Instruments Ltd., Minsk, Belarus). This is a scanning laser microspectrometer coupled to the NIKONTE2000-E inverted microscope (Nikon Corp., Tokyo, Japan) and comprises a picosecond laser source for CARS option and 633 nm He-Ne laser (model 05-LHP-991, Melles Griot, for the Raman and SERS measurements. The laser power at the sample was controlled by a variable neutral filter with the 0–3 optical density.

PL and photoluminescence excitation (PLE) spectra of P3HT in the absence and in the presence of CNTs of the type M + S-SWNTs, S-SWNTs and M-SWNTs were recorded, in the right-angle geometry, with a Fluorolog-3 spectrophotometer, FL3-2.2.1 model, from Horiba Jobin Yvon ((Palaiseau, France).

## 4. Conclusions

In this work, new results concerning the PL properties of the composites based on P3HT and CNTs of the type S-SWNTs and M-SWNTs have been reported. The results reported by UV-VIS-NIR and FTIR spectroscopy, SERS and PL allow to highlight the following conclusions: (i) the chemical interaction of P3HT with CNTs of the type S-SWNTs and M-SWNTs leads to a non-covalently functionalization of CNTs with macromolecular compound; (ii) the electrochemical polymerization of 3HT using the electrode of Au modified with S-SWNTs and M-SWNTs results in both a covalently and non-covalently functionalization of CNTs with P3HT; (iii) a P3HT PL quenching process was demonstrated to be induced by both M-SWNTs and S-SWNTs, this experimental fact was explained taking into account the various de-excitation pathways according to the energy levels diagram of the constituents of the two composite materials; (iv) the *θ*_PL_ values reported in the case of the composites prepared by electrochemical method are lower in contrast with those obtained by chemical interaction of the two constituents; (v) the different *θ*_PL_ value reported in the case of the P3HT/M + S-SWNTs composite, in contrast with P3HT, origins metallic nanotubes. In the case of the P3HT/M-SWNTs composite, prepared by electrochemical method, the *θ*_PL_ value equal to 0° indicates a parallel orientation of the excitation and emission transition dipoles to the basal plane of graphitic lattice of CNTs.

## Data Availability

The data presented in this study are available on request from the corresponding author.

## Sample Availability

Samples of the compounds are available from the authors.
